# PALM: a powerful and adaptive latent model for prioritizing risk variants with functional annotations

**DOI:** 10.1093/bioinformatics/btad068

**Published:** 2023-02-06

**Authors:** Xinyi Yu, Jiashun Xiao, Mingxuan Cai, Yuling Jiao, Xiang Wan, Jin Liu, Can Yang

**Affiliations:** Shenzhen Research Institute of Big Data, Shenzhen 518172, China; Department of Mathematics, The Hong Kong University of Science and Technology, Hong Kong SAR, China; Shenzhen Research Institute of Big Data, Shenzhen 518172, China; Department of Mathematics, The Hong Kong University of Science and Technology, Hong Kong SAR, China; Department of Mathematics, The Hong Kong University of Science and Technology, Hong Kong SAR, China; Department of Biostatistics, City University of Hong Kong, Hong Kong SAR, China; School of Mathematics and Statistics, Wuhan University, Wuhan 430072, China; Shenzhen Research Institute of Big Data, Shenzhen 518172, China; Centre for Quantitative Medicine, Health Services & Systems Research, Duke-NUS Medical School, Singapore 169857, Singapore; School of Data Science, The Chinese University of Hong Kong-Shenzhen, Shenzhen 518172, China; Department of Mathematics, The Hong Kong University of Science and Technology, Hong Kong SAR, China

## Abstract

**Motivation:**

The findings from genome-wide association studies (GWASs) have greatly helped us to understand the genetic basis of human complex traits and diseases. Despite the tremendous progress, much effects are still needed to address several major challenges arising in GWAS. First, most GWAS hits are located in the non-coding region of human genome, and thus their biological functions largely remain unknown. Second, due to the polygenicity of human complex traits and diseases, many genetic risk variants with weak or moderate effects have not been identified yet.

**Results:**

To address the above challenges, we propose a powerful and adaptive latent model (PALM) to integrate cell-type/tissue-specific functional annotations with GWAS summary statistics. Unlike existing methods, which are mainly based on linear models, PALM leverages a tree ensemble to adaptively characterize non-linear relationship between functional annotations and the association status of genetic variants. To make PALM scalable to millions of variants and hundreds of functional annotations, we develop a functional gradient-based expectation–maximization algorithm, to fit the tree-based non-linear model in a stable manner. Through comprehensive simulation studies, we show that PALM not only controls false discovery rate well, but also improves statistical power of identifying risk variants. We also apply PALM to integrate summary statistics of 30 GWASs with 127 cell type/tissue-specific functional annotations. The results indicate that PALM can identify more risk variants as well as rank the importance of functional annotations, yielding better interpretation of GWAS results.

**Availability and implementation:**

The source code is available at https://github.com/YangLabHKUST/PALM.

**Supplementary information:**

Supplementary data are available at *Bioinformatics* online.

## 1 Introduction

Over the past 15 years, genome-wide association studies (GWASs) have greatly deepened our understanding of genetic basis of human phenotypes (Hu *et al.*, 2022; Xiao *et al.*, 2022). As of December 2022, more than 6180 GWASs and 458 000 associations between single nucleotide polymorphisms (SNPs) and human phenotypes have been reported at the GWAS catalog. Despite the fruitful findings from GWASs, much efforts are still needed to address the challenges in GWASs. First, nearly 90% of the genome-wide significant SNPs are located in the non-coding regions (Welter *et al.*, 2014). The molecular processes and pathways through which these SNPs affect complex phenotypes largely remain unclear. It is highly demanding to systematically examine their biological roles. Second, due to the polygenic genetic architectures, the identified genome-wide significant SNPs can only explain a small proportion of heritability (Wray *et al.*, 2018). This implies that many SNPs with small or moderate effects have not been identified. It is highly desired to have reliable statistical methods for risk SNP prioritization.

To address the above problems, valuable information other than GWAS summary statistics should be utilized. Functional annotation serves as a promising source of auxiliary information (Hu *et al.*, 2017). In recent years, large genomics consortia have been making great efforts on creating various functional annotations, including epigenomic maps and gene expression data (Kundaje *et al.*, 2015; The GTEx Consortium, 2020). Emerging functional annotations reveal that SNPs with different genomic features are not equally important. Trait-associated SNPs are often enriched in gene regulatory regions or regions near expressed genes in specific tissues or cell types (Cai *et al.*, 2020; Pickrell, 2014). Key tissues, cell types and regulatory regions associated with diseases can be systematically localized with the knowledge of enrichment pattern (Breeze *et al.*, 2022; Shi *et al.*, 2020).

The rich functional information of human genome and evidence from enrichment analysis provide us with an unprecedented opportunity to (i) prioritize more risk SNPs and (ii) detect trait-relevant cell types or tissues to better understand the biological mechanism of common traits/diseases. In statistics, the two-groups model (TGM) (Efron, 2008) is widely adopted for false discovery rate (FDR) control in the multiple testing problem. In recent years, several methods have been built on the TGM for integrating functional annotations with GWAS summary statistics. To name a few, GPA (Chung *et al.*, 2014) extends the TGM by simultaneously modeling both pleiotropy and functional annotations. FDRreg (Scott *et al.*, 2015) allows the prior of SNP association status to be modulated by covariates through a regression model. Along this direction, a latent sparse mixed model (LSMM) (Ming *et al.*, 2018) further extends the regression model to handle a large number of annotations and detect relevant functional annotations. Very recently, GPA-Tree (Khatiwada *et al.*, 2022) generalizes GPA by using a decision tree to adaptively specify the prior of SNP association status.

Despite the above progress, the existing methods still have their own limitations. First, most existing methods assume a linear relationship between functional annotations and the association status. Ignoring the potential non-linearity may undermine the valuable information embedded in functional annotations and thereby degrade the performance of prioritizing risk SNPs. Although GPA-Tree adopts the decision tree algorithm to characterize the potential non-linearity, a single decision tree often cannot fully capture the relationship between functional annotations and association status. In addition, a single decision tree is known to be not very stable (Breiman, 2001). This may lead to an unsatisfactory control of FDR. Second, most existing methods, e.g. GPA and FDRreg, were designed to integrate a small number of functional annotations. They may not be able to scale up to a large number of functional annotations in integrative analysis. New statistical methods are highly demanding to address these limitations.

In this article, we propose a powerful and adaptive latent model (PALM), to integrate GWAS summary statistics with functional annotations. Unlike existing methods, PALM uses a tree ensemble as the non-linear model to characterize the relationship between functional annotations and the association status. To make PALM scalable to hundreds of annotations and millions of genetic variants, we develop a functional gradient-based expectation–maximization (EM) algorithm, where the posterior of SNP association status is evaluated at the E-step, and a new tree is added into the model in the M-step by a boosting strategy (Friedman, 2001). In such a way, our model can become more and more flexible, resulting in a stable improvement over existing methods. Through comprehensive simulations, we demonstrate that PALM can not only well control false positive rate but also significantly improve the statistical power of prioritizing risk SNPs over the existing methods. We then apply PALM to prioritize risk SNPs of 30 GWASs by integrating 127 cell-type-specific functional annotations and illustrate that PALM outperforms compared methods in most GWASs. In addition, with the boosted tree algorithm and the regularization strategy, PALM can handle missing values and shows its robustness. Moreover, PALM can automatically rank the relative importance of functional annotations, offering more interpretable biological results.

## 2 Materials and methods

### 2.1 Powerful and adaptive latent model

Suppose we have performed hypothesis testing to examine whether a SNP is associated with a given phenotype in GWAS and obtained the *P*-values of genome-wide SNPs $\left\{p_{1},p_{2},\ldots ,p_{M}\right\}$, where *M* is the number of SNPs. We introduce a latent variable $Z_{j}\in \{0,1\}$ to indicate the association status of the *j*-th SNP. We consider a TGM, where the *P*-value of each SNP is either from a null group ($Z_{j}=0$) or a non-null group ($Z_{j}=1$) according to its association status:

(1) $$\begin{array}{cc} \text{Null group}\;(Z_{j}=0): & \;p_{j}∼U(0,1),\\ \text{Non}-\text{null group}\;(Z_{j}=1): & \;p_{j}∼\mathrm{Beta}(\alpha ,1). \end{array}$$

The above TGM assumes that *P*-values from the null group follow the uniform distribution U(0,1) and *P*-values from the non-null group follow the beta distribution with shape parameter α and 1, where 0<α<1 is used to model the fact that *P*-values tend to be closer to 0 for associated SNPs. In the basic TGM, the prior probabilities of latent variable are common for all the SNPs: $\pi_{0}\;:=\text{Pr}(Z_{j}=0),\;\pi_{1}\;:=\text{Pr}(Z_{j}=1),j=1,\ldots ,M$ (Efron, 2008). Thus, the determination of SNP association status only relies on the ‘direct’ evidence—*P*-values from GWAS summary statistics. In other words, all the SNPs are treated with equal prior. However, SNPs are actually not equally important and SNPs with biological functions tend to be enriched in GWAS signals (Schork *et al.*, 2013). Functional annotations from the concerted efforts of large genomic consortia provide ‘indirect’ evidence to determine SNP association status. Therefore, it is an exciting opportunity to combine the functional annotations as indirect evidence with the direct evidence (*P*-values from GWAS) to increase the power of prioritizing risk SNPs and offer more biologically interpretable GWAS results.

Suppose we have collected annotations in a matrix $A\in R^{M\times L}$, where *L* is the number of functional annotations, entry $A_{j,l}$ corresponds to the annotation status of the *j*-th SNP given by the *l*-th functional annotation. In the simplest case, $A_{j,l}$ is binary, where $A_{j,l}=1$, and $A_{j,l}=0$ means that SNP *j* can be active or inactive according to the *l*-th functional annotation, respectively. In our formulation, we allow $A_{j,l}$ to be a continuous variable. For example, a higher value in $A_{j,l}$ can indicate SNP *j* is more likely to have a functional role. To model the relationship between functional annotations and SNP association status, we assume that the prior of SNP *j*’s association status can be modulated by its functional role as $\pi_{j0}=\text{Pr}(Z_{j}=0|A_{j})$ and $\pi_{j1}=\text{Pr}(Z_{j}=1|A_{j})$, where $A_{j}$ is the *j*-th row of the annotation matrix corresponding to the *j*-th SNP, j=1,…,M. More specifically, we relate the association status $Z_{j}$ with $A_{j}$ through the logit link as:

(2) $$\text{log}\;\frac{\text{Pr}(Z_{j}=1|A_{j})}{\text{Pr}(Z_{j}=0|A_{j})}=F(A_{j}),$$

where *F* can be a linear or non-linear function. For example, LSMM (Ming *et al.*, 2018) and FDRreg (Scott *et al.*, 2015) choose *F* to have a linear form, $F(A_{j})=\beta_{0}+A_{j}\beta$. However, such a model is limited to the linear relationship between the association status and the annotations in the logit scale. In real data analysis, functional annotations may influence the SNP association status in a much more complicated way (Przybyla and Gilbert, 2022). As the number of SNPs is usually more than 1 million, it gives us an opportunity to learn a more complex model structure than linear models.

To achieve this goal, we assume that *F* in Equation (2) is represented by a tree ensemble:

(3) $$F(A_{j})=f_{0}+\sum_{t=1}^{T}f_{t}(A_{j}),$$

where $f_{t}$ is a regression tree with depth *D*, t=1,2,…,T, and *T* is the total number of trees. The advantages of the proposed model are threefold. First, tree ensembles are able to capture more flexible relationship between functional annotations and SNP association status. Second, the proposed model naturally inherits several salient features of regression trees (Breiman *et al.*, 1984), such as ranking variable importance and handling missing values. Third, we can develop an efficient algorithm to estimate the non-linear model from data, and make it scalable to large-scale real data analysis.

### 2.2 Algorithm

It is worthwhile to note that existing boosted tree algorithms cannot be directly applied here and a stable fitting of the function *F* is not an easy task. This is because they are supervised learning algorithms and thus require the response $Z_{j}$ in Equation (2) to be known. In our formulation, however, $Z_{j}$ is unknown. Therefore, we need a new algorithm to obtain the tree ensemble in the presence of latent variables.

To do so, we write down the probabilistic model of the complete data based on Equations (1) and (2):

(4) $$\text{Pr}(p,Z|A;F,\alpha )=\prod_{j=1}^{M}\pi_{j0}^{1-Z_{j}}{\left(\pi_{j1}\phi (p_{j};\alpha )\right)}^{Z_{j}},$$

where $p={\left[p_{1},\ldots ,p_{M}\right]}^{T}$ and $Z={\left[Z_{1},\ldots ,Z_{M}\right]}^{T}$ are the vectors of *P*-values and latent variables for *M* SNPs, respectively, $\phi (p;\alpha )=\alpha p^{\alpha -1}$ is the density function of Beta(α,1), $\pi_{j1}=1/(1+\text{exp}(-F(A_{j})))$ and $\pi_{j0}=1-\pi_{j1}$. Marginalizing over the latent variables Z, the probabilistic model of the observed *P*-values becomes:

(5) $$\begin{array}{c} \text{Pr}(p|A;F,\alpha )=\prod_{j=1}^{M}\sum_{Z_{j}\in \{0,1\}}\pi_{j0}^{1-Z_{j}}{\left(\pi_{j1}\phi (p_{j};\alpha )\right)}^{Z_{j}}\\ =\prod_{j=1}^{M}[\pi_{j0}+\pi_{j1}\phi (p_{j};\alpha )]. \end{array}$$

Then, we have the marginal log-likelihood function:

(6) $$\text{log}\;\;\text{Pr}(p|A;F,\alpha )=\sum_{j=1}^{M}\;\text{log}\;[\pi_{j0}+\pi_{j1}\phi (p_{j};\alpha )].$$

Our goal is to fit the tree ensemble *F* and estimate α by maximizing the marginal log-likelihood given in Equation (6). To achieve this goal, we propose a new algorithm, which combines the EM algorithm with the tree boosting algorithm (Chen and Guestrin, 2016; Friedman, 2001). In the E-step of the (t+1)-th iteration,

$$\begin{array}{c} \begin{array}{cc} & Q(F,\alpha |F^{\left(t\right)},\alpha^{\left(t\right)})\\ = & E_{Z|p;F^{\left(t\right)},\alpha^{\left(t\right)}}[\text{log}\;\;\text{Pr}(p,Z|A;F,\alpha )]\\ = & E_{Z|p;F^{\left(t\right)},\alpha^{\left(t\right)}}\sum_{j=1}^{M}[(1-Z_{j})\;\text{log}\;\pi_{j0}+Z_{j}(\text{log}\;\pi_{j1}+\text{log}\;\phi (p_{j};\alpha ))]\\ = & \sum_{j=1}^{M}[q_{j0}^{\left(t\right)}\;\;\text{log}\;\;\pi_{j0}+q_{j1}^{\left(t\right)}\;(\text{log}\;\;\pi_{j1}+\text{log}\;\;\alpha +(\alpha -1)\;\;\text{log}\;\;p_{i})], \end{array} \end{array}$$

where

$$\begin{array}{c} \begin{array}{c} q_{j1}^{\left(t\right)}=\text{Pr}(Z_{j}=1|p_{j},A_{j};F^{\left(t\right)},\alpha^{\left(t\right)})\\ =\frac{\pi_{j1}\text{Pr}(p_{j}|Z_{j}=1;F^{\left(t\right)},\alpha^{\left(t\right)})}{\pi_{j0}\text{Pr}(p_{j}|Z_{j}=0;F^{\left(t\right)},\alpha^{\left(t\right)})+\pi_{j1}\text{Pr}(p_{j}|Z_{j}=1;F^{\left(t\right)},\alpha^{\left(t\right)})}, \end{array} \end{array}$$

and $q_{j0}^{\left(t\right)}=1-q_{j1}^{\left(t\right)}$.

In the M-step of the (t+1)-th iteration, we aim to increase the *Q* function w.r.t. α and *F*. By solving $\frac{\partial Q}{\partial \alpha }=0$, we have a closed form solution to update α as

$$\alpha^{\left(t+1\right)}=-\frac{\sum_{j=1}^{M}q_{j1}^{\left(t\right)}}{\sum_{j=1}^{M}q_{j1}^{\left(t\right)}\;\text{log}\;p_{j}}.$$

Then, we update *F* using the tree boosting strategy as $F^{\left(t+1\right)}=F^{\left(t\right)}+\nu f_{t+1}$, where ν∈(0,1) is the shrinkage parameter (Friedman, 2001). To find $f_{t+1}$, we approximate the *Q* function by its second-order Taylor expansion:

$$\begin{array}{c} Q(f_{t+1}|F^{\left(t\right)})\\ =\sum_{j=1}^{M}[q_{j1}^{\left(t\right)}f_{t+1}(A_{j})-\text{log}\;(1+e^{F^{\left(t\right)}(A_{j})+f_{t+1}(A_{j})})]+\mathrm{const}\\ ≃\sum_{j=1}^{M}[g_{j}f_{t+1}(A_{j})+\frac{1}{2}h_{j}f_{t+1}{\left(A_{j}\right)}^{2}]+\mathrm{const}, \end{array}$$

where the first and second derivatives are given by:

$$\begin{array}{c} g_{j}=\frac{\partial Q}{\partial f_{t+1}}{|}_{f_{t+1}(A_{j})=0}=q_{j1}^{\left(t\right)}-\frac{1}{1+\text{exp}(-F^{\left(t\right)}(A_{j}))},\\ h_{j}=\frac{\partial^{2}Q}{\partial f_{t+1}^{2}}{|}_{f_{t+1}(A_{j})=0}=-\frac{\;\text{exp}(-F^{\left(t\right)}(A_{j}))}{{\left(1+\text{exp}(-F^{\left(t\right)}(A_{j}))\right)}^{2}}. \end{array}$$

With the data ${\left\{A_{j},-\frac{g_{j}}{h_{j}}\right\}}_{j=1}^{M}$, we fit a new regression tree ${\hat{f}}_{t+1}$ by solving the optimization problem:

(7) $${\hat{f}}_{t+1}=\underset{f_{t+1}}{\text{argmax}}\sum_{j=1}^{M}\frac{1}{2}h_{j}{\left[-\frac{g_{j}}{h_{j}}-f_{t+1}(A_{j})\right]}^{2}.$$

Then, the tree ensemble becomes:

$$F^{\left(t+1\right)}(A_{j})=F^{\left(t\right)}(A_{j})+\nu {\hat{f}}_{t+1}(A_{j}).$$

Accordingly, the prior of SNP association status is updated as:

$$\pi_{1j}^{\left(t+1\right)}=\frac{1}{1+\text{exp}(-F^{\left(t+1\right)}(A_{j}))}.$$

Clearly, information in functional annotations is gradually built in to modulate the prior of SNP association status. The marginal log-likelihood given in Equation (6) can be increased in each EM step and the convergence of EM algorithm is guaranteed.

### 2.3 Regularization and missing values

For PALM, the regularization is determined by the combination of the number of trees and the shrinkage parameter. Recall that a new tree is fitted into our model in each M-step of the EM algorithm. To determine the optimal number of trees, we use *K*-fold cross-validation, where we choose K=5 as the default setting. Then, we fit model again on the entire dataset and obtain the final model based on the optimal number of trees determined by cross-validation. For PALM, the shrinkage parameter ν∈(0,1) can be used to reduce the impact of each tree and it is also known as the ‘learning rate’. A smaller value of ν typically improves model stability and has better generalization ability (Friedman, 2001). We choose ν=0.1 as the default setting.

One important feature of PALM is its ability to handle missing values. In general, there are two common approaches to deal with missing values for tree-based methods. The first approach is choosing a direction for ‘missing’. The second approach is constructing a series of surrogate splits for each node (Hastie *et al.*, 2009). In PALM implementation, we utilize the XGBoost package, which handles missing values with the first approach. Specifically, a default direction is added to each tree node in the training stage. During the testing stage, if one SNP misses an annotation, it will be classified into the default direction of the corresponding node. Importantly, the default directions are learnt from the data by the sparsity-aware split finding approach rather than pre-fixed (Chen and Guestrin, 2016).

### 2.4 Identifying risk SNPs with FDR control and ranking the importance of functional annotations

With the fitted model, we can obtain the estimated parameter $\hat{\alpha },{\hat{\pi }}_{j1}=\frac{1}{1+\text{exp}(-F^{\left(T\right)}(A_{j}))},{\hat{\pi }}_{j0}=1-{\hat{\pi }}_{j1}$ and posterior probability $\text{Pr}(Z_{j}=1|p_{j},A_{j};F^{\left(T\right)},\hat{\alpha })=\frac{{\hat{\pi }}_{j1}\phi (p_{j};F^{\left(T\right)},\hat{\alpha })}{{\hat{\pi }}_{j0}+{\hat{\pi }}_{j1}\phi (p_{j};F^{\left(T\right)},\hat{\alpha })}$. Given its *P*-value and annotation vector, the local FDR of the *j*-th SNP can be estimated as: ${\hat{\text{fdr}}}_{j}:=\text{Pr}(Z_{j}=0|p_{j},A_{j};\hat{\alpha })=1-\text{Pr}(Z_{j}=1|p_{j},A_{j};\hat{\alpha })$. We control the global FDR by direct posterior probability approach (Newton *et al.*, 2004). Specifically, we first sort the estimated local FDR in an ascending order: ${\hat{\text{fdr}}}_{\left(1\right)}\leq {\hat{\text{fdr}}}_{\left(2\right)}\leq \ldots \leq {\hat{\text{fdr}}}_{\left(M\right)}$, then find the largest *k* satisfying: ${\hat{\text{Fdr}}}_{\left(k\right)}\equiv \frac{\sum_{j=1}^{k}{\hat{\text{fdr}}}_{\left(j\right)}}{k}\leq \tau$, where τ is the pre-specified global FDR control level, e.g. τ=0.1. Finally, SNPs whose order is smaller than or equal to *k* will be declared to be associated with the phenotype.

Functional annotations may not be equally important for prioritization of risk SNPs. Recall that the importance of a variable ranked in the tree algorithm is given by the total reduced error when a node of the tree is splitted on this variable. The more error reduced by splitting on a variable, the more important of the variable is. By inheriting the merit of regression trees, the model given by PALM can be used to rank the importance of functional annotations. Specifically, the variable importance of the *l*-th annotation is given by

(8) $$I_{l}=\frac{1}{T}\sum_{t=1}^{T}I_{t,l},$$

where $I_{t,l}$ is the importance of the *l*-th annotation evaluated at the *t*-th tree. With the importance of functional annotations, PALM’s output is very helpful for biologically meaningful interpretation of GWAS results.

## 3 Results

### 3.1 Simulation study

We conducted comprehensive simulation studies to gauge the performance under different function *F* and signal parameters. First, we generated *P*-values of the null group from uniform distribution U(0,1). For *P*-values of the non-null group, we used a ‘bimodal’ distribution: $\mu_{j}∼0.48N(-2,1)+0.04N(0,16)+0.48N(2,1)$. Then *z*-score $z_{j}$ was generated by adding a random noise to $\mu_{j}$: $z_{j}∼N(\mu_{j},1)$, and the corresponding *P*-value was calculated by the tail probability of $z_{j}$: $p_{j}=2(1-\Phi (|z_{j}|))$, where Φ is the cdf of N(0,1). Clearly, the *P*-values from the non-null group are different from the beta distribution given in our model [Equation (1)]. The simulation here is designed to evaluate the robustness of our proposed method in the presence of model misspecification.

To determine whether the *j*-th *P*-value was from the null group or the non-null group, we assumed that the probability for the non-null group $\pi_{j1}$ was specified as

(9) $$\pi_{j1}=\frac{1}{1+\text{exp}(-F(A_{j}))},$$

and the prior probability for the null group was $\pi_{j0}=1-\pi_{j1}$. For the true function *F*, to examine the performance of PALM in multiple aspects and compare it with other methods, we consider five cases:

(10) $$\begin{array}{l} (A)\;F=-3,\\ (B)\;F(A_{j,1},A_{j,2})=-3+1.5A_{j,1}+1.5A_{j,2},\\ (C)\;F(A_{j,1})=-4.25+2A_{j,1}^{2}+2A_{j,2}^{2}-2A_{j,1}A_{j,2},\\ \begin{array}{c} \left(D)\;F(A_{j,1},A_{j,2},A_{j,3},A_{j,4},A_{j,5})=-4+4\;\text{sin}(\pi A_{j,1}A_{j,2}\right)\\ +2{\left(A_{j,3}-A_{j,4}\right)}^{2}+A_{j,4}+0.5A_{j,5},\\ (E)\;F(A_{j,1},A_{j,2})=\{\begin{array}{cc} 1-6A_{j,1}^{2} & \text{if}\;A_{j,2}=0,\\ -1+2A_{j,1}-6A_{j,1}^{2} & \text{if}\;A_{j,2}=1. \end{array} \end{array} \end{array}$$

Case (A) serves as a negative control, where all annotations are irrelevant; Case (B) is a linear relationship with two relevant annotations; Case (C) is a simple quadratic function with interaction among two annotations; Case (D) is a more complicated function with a quadratic term and a sinusoidal term involving five relevant annotations; and Case (E) is a case function involving interaction between a continuous annotation $A_{:,1}$ and a binary annotation $A_{:,2}$. We generated annotation matrix A whose entries were from uniform distribution U(−1,1). For Case (E), first we generated a categorical vector for $A_{:,2}$ and then specified $\pi_{j1}$ by Equation (9) and generated the association status $Z_{j}∼\mathrm{Bernoulli}(\pi_{j1}),j=1,\ldots ,M$.

We set the number of SNPs $M\in \{2\times 10^{4},5\times 10^{4},1\times 10^{5}\}$ and the number of annotation variables L∈{50,100}. Methods in comparison include three methods using only GWAS summary statistics: two-groups model of *P*-values (TGM-Pval), two-groups model of *z*-scores (TGM-Zval) and the Benjamini–Hochberg (BH) procedure, and three methods integrating functional annotations with GWAS results: LSMM, GPA-Tree and PALM. Here, we considered fitting PALM with Tree depths 1 and 2, denoted as PALM-D1 and PALM-D2, respectively. PALM-D1 can characterize the non-linear relationship with additive models, and PALM-D2 is a more flexible non-linear model by allowing interaction among annotations. For each method, we use the default parameter setting. We controlled global FDR at the nominal level 0.1, and evaluated the empirical FDR as the fraction of falsely identified SNPs among all the identified SNPs and statistical power as the fraction of correctly identified SNPs in the non-null group of each method.

**Fig. 1.**
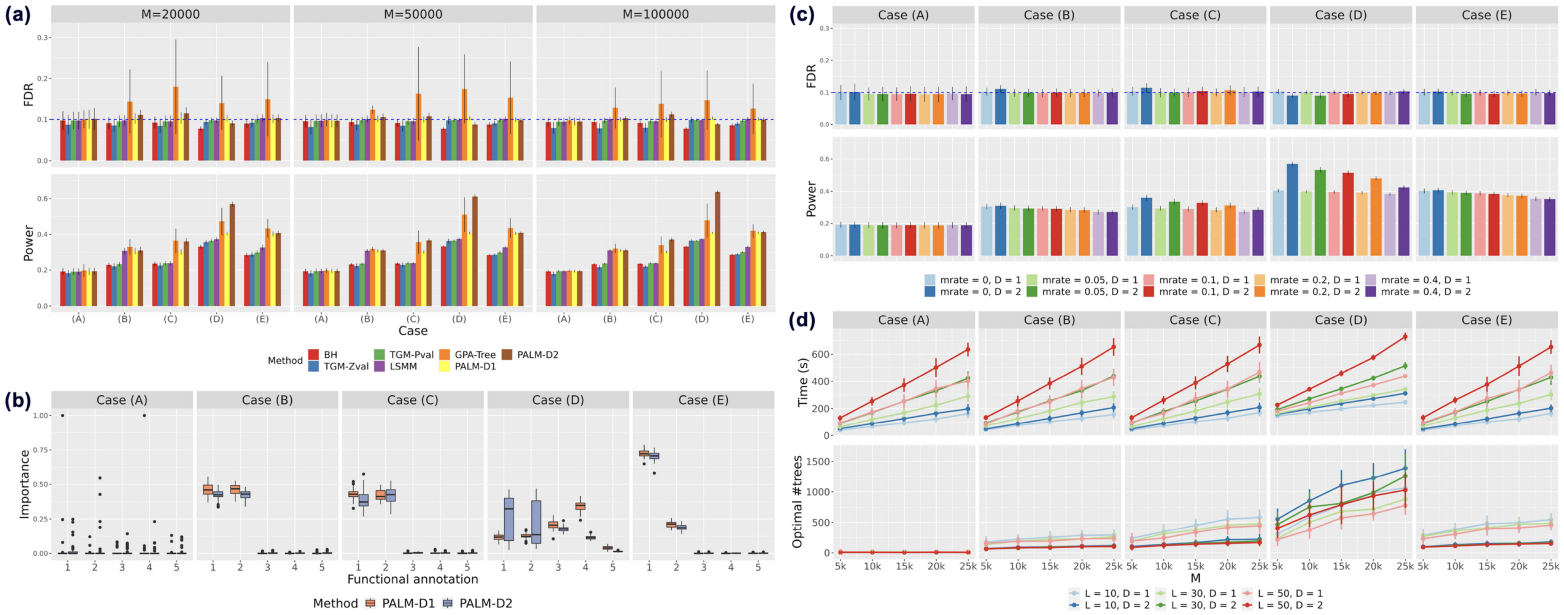
Simulation results. (**a**) The comparison of PALM-D1 and PALM-D2 with other related methods, including BH, TGM-Pval, TGM-Zval, LSMM and GPA-Tree. The number of SNPs *M* varied at $\left\{2\times 10^{4},5\times 10^{4},1\times 10^{5}\right\}$ and the number of annotations L=100. (**b**) Relative importance of the first five annotations by PALM. (**c**) Performance of PALM under different missing value rates of functional annotations. For (b) and (c), the number of SNPs M=20 000 and the number of annotations L=50. (**d**) Computational time and optimal number of trees of PALM. We varied the number of SNPs *M* and the number of annotations *L* with CV folds K=2

Figure 1a shows the comparison of PALM with the above methods. One can see that FDR was well controlled at the nominal level (τ=0.1) in all scenarios for both PALM-D1 and PALM-D2. Except GPA-Tree, all the compared methods controlled their FDR at the nominal level. The unsatisfactory FDR control of GPA-Tree could be attributed to the instability of a single tree (Breiman, 1996). When all the annotations were irrelevant to the association status of SNPs [Case (A)], methods integrating annotations had almost the same power with the standard BH procedure. This is a desired property, indicating that these integrating methods do not overuse annotations when they are irrelevant. When the relationship was of a linear form [Case (B)], methods integrating annotations had a significant gain in statistical power compared with methods only using summary statistics. This case illustrates the benefit from incorporating annotation information. Here, PALM achieved comparable power with LSMM which was designed for modeling linear relationship, indicating that PALM did not overfit despite its flexibility. In the presence of both non-linearity and two-way interactions [Case (C)], PALM-D2 was the winner as expected. PALM-D1 outperformed LSMM because it can model non-linearity while LSMM cannot. For Case (D), PALM-D2 outperformed other methods again. The superiority of PALM-D2 became clearer in the increasing trend of *M*, as the model can be better fitted with a larger number of SNPs. In this scenario, there was a notable gap between the power of GPA-Tree and PALM-D2, indicating that a single decision tree could not accurately capture some complicated relationship between association status and annotations. For Case (E), the power of PALM and GPA-Tree was roughly matched, dominating other methods but GPA-Tree tended to produce more false positives. In summary, PALM remarkably increased statistical power for various relationship between annotations and association status. We also conducted additional simulations with alternative *z*-score distribution shapes, i.e. ‘big-normal’, ‘near-normal’, ‘skew’ and ‘spiky’. The patterns of FDR control and statistical power for all the compared methods are similar to Figure 1a. Details can be found in Supplementary Figures S1–S4. In GWAS, the *z*-scores of SNPs are typically calculated from a linear model with individual data. We further investigate the performance of these methods under the setting where *z*-scores are obtained from linear regression with simulated genotype and a realistic heritability. The patterns of FDR and power are similar to Figure 1a (see Supplementary Fig. S5), validating the effectiveness of PALM with *z*-scores generated from a linear model.

PALM can automatically rank relevant annotations. Figure 1b shows the relative variable importance evaluated by PALM [Equation (8)]. In Case (A) with no enriched annotations, the relative importance of all annotations was evaluated to be null. In other words, no annotation was assessed to be relevant in prioritizing risk variants, explaining why PALM had the same power with BH procedure in this scenario. In Case (B) where each of the two relevant annotations took half of the contribution to the prior probability, the variable importance assessed by PALM was consistent with the function design [Equation (10)]. For Cases (C) and (E), PALM also correctly ranked the importance of functional annotations. Note that in Case (D), due to the different tree depths, the importance ranked by PALM-D1 and PALM-D2 are different. Theoretically, trees with Depth 2 can model interactions but trees with Depth 1 cannot. Hence the importance ranked by PALM-D2 is supposed to be more accurate than that by PALM-D1. Since PALM-D1 cannot model interactions and $A_{1}$, $A_{2}$ only appear together while $A_{3}$, $A_{4}$ have independent terms, it is reasonable that PALM-D1 underestimates the importance of $A_{1}$, $A_{2}$, making $A_{3}$, $A_{4}$ look more importance. Moreover, PALM can quantify interaction effects between two annotations using Friedman’s H-statistic (Friedman and Popescu, 2008). Details about H-statistic and pairwise interaction estimation of the first five annotations in Cases (B–E) can be found in Supplementary Section S1.3.

Compared with other existing methods, a unique property of PALM is its superior ability to handle missing values in functional annotations. By taking advantage of the XGBoost implementation, PALM is able to handle missing values by the sparsity-aware split finding strategy (Chen and Guestrin, 2016). To evaluate the influence of missing values in the annotation matrix on the performance of PALM, we conducted simulations under different missing value rates, i.e. mrate∈{0.05,0.1,0.2,0.4}. Figure 1c shows that missing value rates have little influence on FDR control. For the statistical power, it is not affected by missing values in Case (A) when no annotation was enriched. In other cases where some annotations were enriched, the statistical power gradually decreased when missing value rate increased due to the loss of annotation information. However, a small fraction of missing values (e.g. 5% and 10%) had a very minor effect on the performance of PALM. Even when 40% of the annotations were missing, the power were still higher than methods without integrating annotations, suggesting that PALM was able to efficiently utilize available annotations to improve risk variants prioritization. Similar conclusion about the influence of missing value rates can be drawn for other *z*-score distributions (Supplementary Figs S7–S10). To our best knowledge, other methods cannot handle the missing value issue in a proper way.

The computational time of PALM mainly depends on the CV folds *K*, the tree depth *D*, the number of variants *M* and the number of annotations *L*. Figure 1d shows that with the same CV folds and tree depth, the computational time is roughly linear with *M* and *L* in all scenarios. For the optimal number of trees, it generally increases with *M* and decreases with *D* in the same trend of overfitting risk. Besides, the optimal number of trees is closely related to the relationship between the association status and annotations. For Case (A), only a small number of trees in the final model are allowed; for Cases (B), (C) and (E) with relatively simple non-linear relationships, PALM-D2 has fewer trees than PALM-D1 as PALM-D2 is more prone to overfitting; for Case (D), PALM-D2 is assigned with more trees than PALM-D1 to better learn the relatively complicated non-linear relationship. This adaptive regularization mechanism helps PALM well control FDR and improves statistical power.

PALM shows great robustness under different hyper-parameter settings. First, by applying PALM with 2-fold CV and 5-fold CV to the same simulated data, we find that the FDR and power are almost the same under different scenarios for both PALM-D1 and PALM-D2 (Supplementary Fig. S14). Second, the shrinkage parameter ν has little influence on the performance of PALM. However, it has some impact on the number of trees of the final model after cross-validation (Supplementary Fig. S15). In particular, a very small shrinkage parameter (e.g. ν=0.01,0.05) will lead to a larger number of trees in the final model, thus more time-consuming. The default shrinkage ν=0.1 is chosen as it can well control FDR and achieves great power with a reasonable computational cost. Third, even with Tree depth 3 or 4, PALM does not suffer from severe FDR inflation (Supplementary Fig. S16).

### 3.2 Real data analysis

In the real data analysis, we integrated summary statistics from 30 GWASs (given in Supplementary Table S4) with 9 genic category annotations and 127 cell-type-specific functional annotations. The genic category annotations includes: upstream, downstream, exonic, intronic, ncRNA exonic, ncRNA intronic, UTR3, UTR5 and intergenic. The cell-type-specific functional annotations are from GenoSkylinePlus (Lu *et al.*, 2017). Each entry in the cell-type-specific annotation matrix is a binary variable indicating whether one SNP has biological function in a specific cell type. To avoid unusually large GWAS signals in the MHC region (Chromosome 6, 25–35 Mb), we excluded SNPs in this region.

**Fig. 2.**
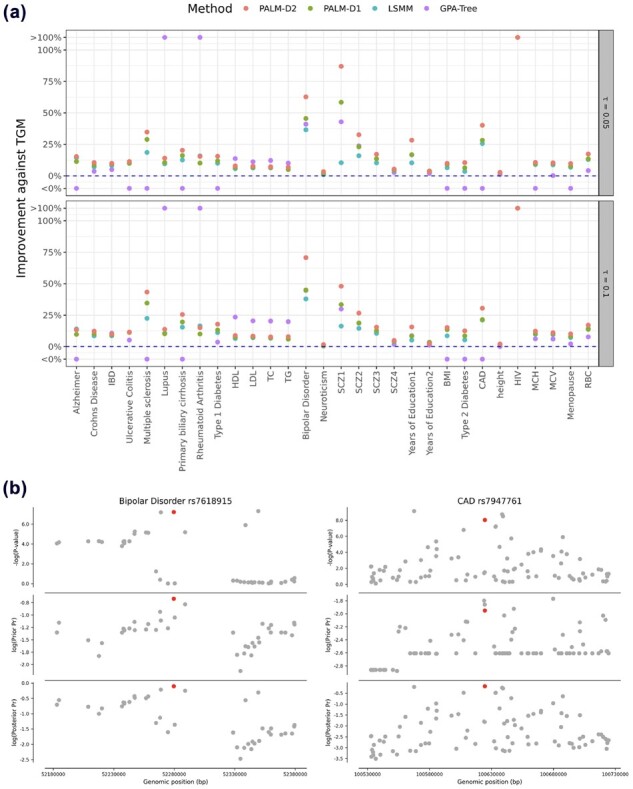
Real data analysis results. (**a**) The improvement on the number of prioritized risk SNPs for PALM-D2, PALM-D1, GPA-Tree and LSMM compared with TGM under the global FDR threshold τ=0.05 and τ=0.1. (**b**) The −log(P)-value, prior and posterior probability of example SNPs prioritized only by PALM-D2 and other SNPs within 100 kb

We compared the power of risk variants prioritization using TGM-Pval, LSMM, GPA-Tree, PALM-D1 and PALM-D2. Figure 2a shows the improvement of PALM-D2, PALM-D1, GPA-Tree and LSMM against TGM. In general, more risk SNPs can be identified using PALM than LSMM, GPA-Tree and TGM (numbers of prioritized risk SNPs are given in Supplementary Tables S2 and S3). It turns out that GPA-Tree does not perform very well. In several GWASs, the number of prioritized SNPs by GPA-Tree was either even less than TGM or much larger than PALM-D2, which may be attributed to the instability of a single tree. Discussion on the issue of GPA-Tree is in Supplementary Section S2.1. We will exclude GPA-Tree from the later discussion. Besides, we have the following observations. First, integrating annotations in SNP prioritization can greatly increase statistical power. The amounts of SNPs identified by PALM and LSMM dominated those by TGM for all the GWASs, confirming that annotation enrichment in risk SNPs is pervasive. Under the global FDR threshold τ=0.1, PALM-D1 and PALM-D2 achieved at least 10% improvement on 17 and 22 GWASs, respectively. Second, PALM-D1 identified more risk SNPs than LSMM for the majority of phenotypes, suggesting that the relationship between annotations and the association status may not be simply expressed as linear in the logit scale. For instance, under τ=0.1, 789 SNPs were identified by PALM-D1 compared with 718 SNPs by LSMM for multiple sclerosis; 451 SNPs were identified by PALM-D1 compared with 429 SNPs by LSMM for bipolar disorder. On the whole, PALM-D1 identified more risk SNPs than LSMM in 25 and 22 GWASs under τ=0.05 and τ=0.1, respectively. Third, the overall performance of PALM-D2 is superior to PALM-D1, which is an extra gain from modeling interaction among annotations. We also perform PALM with Depths 3 and 4 on real data. The result is shown in Supplementary Figure S21.

Some of the SNPs prioritized under τ=0.1 only by PALM-D2 but not by LSMM or TGM have been reported in other studies. Let us take several diseases/traits for examples. In the type 2 diabetes (T2D) GWAS, rs12945601 and rs552707 detected only by PALM-D2 were identified in larger GWASs (Mahajan *et al.*, 2022; Xue *et al.*, 2018). For lipid traits including high density lipoprotein (HDL), low density lipoprotein (LDL) and their closely related disease—coronary artery disease (CAD), rs799160 and rs892161 identified only by PALM-D2 were confirmed to be HDL-associated SNP and LDL-associated SNP, respectively (Klarin *et al.*, 2018; Sinnott-Armstrong *et al.*, 2021); risk SNP rs7947761 reported by PALM-D2 was confirmed by a recent CAD GWAS (Van Der Harst and Verweij, 2018). For autoimmune diseases, multiple sclerosis risk SNP rs6911131, Crohn’s disease risk SNP rs11641184 and lupus risk SNP rs9782955 identified by PALM-D2 were found to be associated with the corresponding diseases (Bentham *et al.*, 2015; International Multiple Sclerosis Genetics Consortium, 2019; Liu *et al.*, 2015). For bipolar disorder, PALM-D2 risk SNP rs7618915 was reported in a meta-analysis study (Chen *et al.*, 2013). We take two SNPs mentioned above to visualize how the functional annotations help to prioritize SNPs (Fig. 2b). Bipolar disorder risk SNP rs7618915, an upstream SNP, is annotated by the important annotations including Monocytes-CD14+ RO01746 Primary Cells, Brain Anterior Caudate and Primary B cells from peripheral blood, which contributes to its high prior probability. Its posterior probability is given by combining its functional prior and small *P*-values. For CAD risk SNP rs7947761, it is an intronic SNP annotated by the important annotations, such as Lung and Fetal Heart. Although it neither has the smallest *P*-value nor prior probability, the combination of the two results in the highest posterior probability amongst the nearby SNPs.

We compared the performances of TGM, LSMM and PALM-D2 on schizophrenia (SCZ) and years of education. The sample sizes of the four SCZ GWASs increase successively (SCZ1: *n* = 17 115 SCZ2: *n*=21 856 SCZ3: *n*=32 143 and SCZ4: *n* = 150 064). In any of the four GWASs, PALM-D2 prioritized more risk SNPs compared with TGM and LSMM while the majorities of SNPs prioritized by TGM, LSMM or PALM-D2 are in common (Supplementary Figs S23 and S24). This suggests that PALM-D2 can not only identify most of the SNPs prioritized without utilizing functional annotations but also additional SNPs failed to be prioritized by TGM or LSMM. Moreover, most of SNPs prioritized by PALM-D2 but not by TGM in a smaller GWAS are recapitulated in the set of SNPs prioritized by TGM in a larger GWAS. For examples, under the global FDR threshold 0.1, PALM-D2 prioritized 1806 additional SNPs not identified by TGM in SCZ3 while 1049 of them can be detected by TGM in SCZ4 (Supplementary Figs S25 and S26). The above observations also hold for 2 years of education GWASs with different sample sizes (Supplementary Figs S27 and S28).

**Fig. 3.**
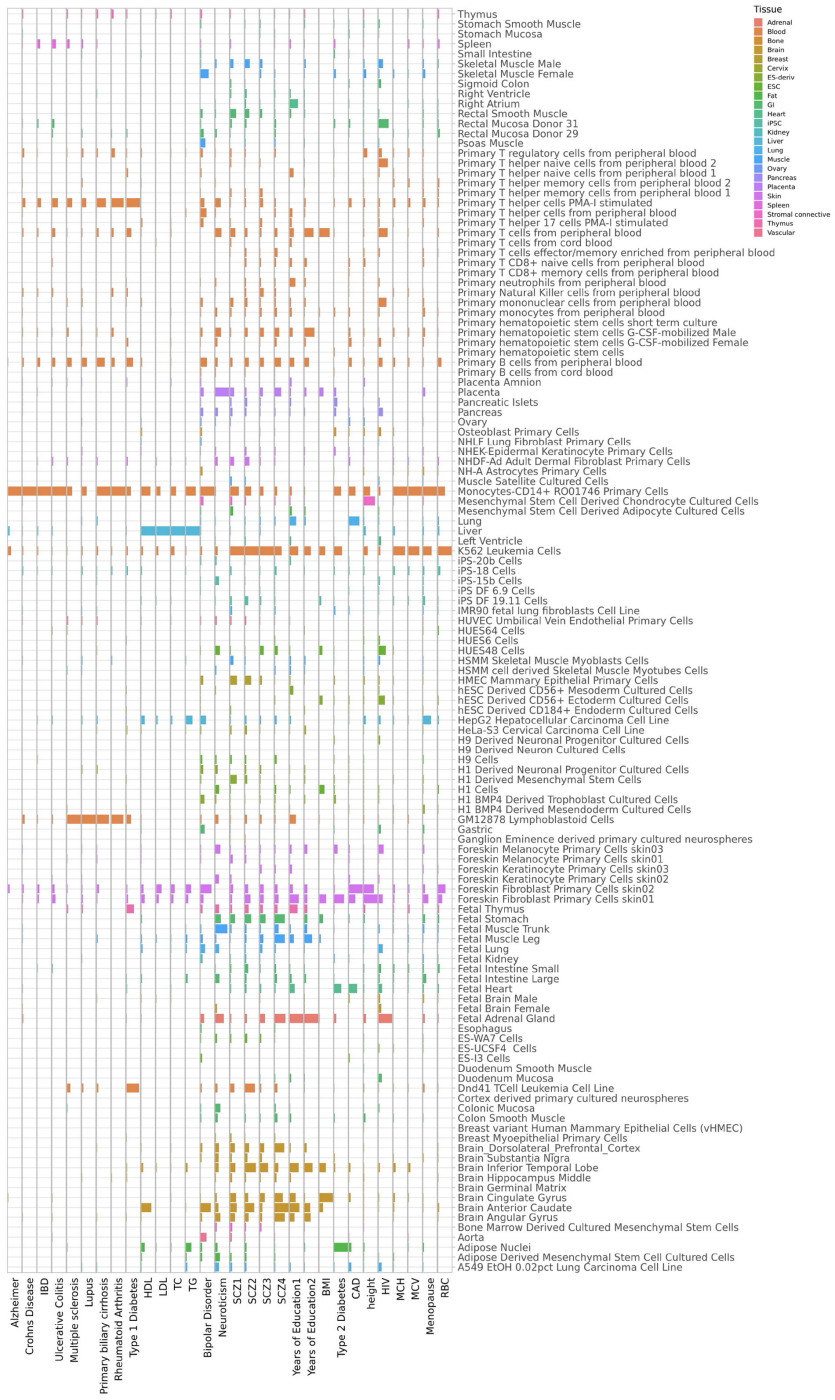
The relative importance of 127 cell-type-specific functional annotations for 30 GWASs evaluated by PALM-D2. Each column represents the standardized importance across annotations in prioritizing risk SNPs of the GWAS where higher bar corresponds to higher importance of the annotation. The column scales are not uniform across phenotypes

Figure 3 shows the relative importance of cell-type-specific annotations ranked by PALM-D2. For autoimmune diseases, multiple immune cells are found relevant. In particular, Monocyte CD14+ primary cells play a dominant role in Alzheimer, Crohn’s disease, inflammatory bowel disease and ulcerative colitis. CD14+ cells were reported to play an essential role in inflammation and infection, which contribute to the development of the autoimmune diseases (Ziegler-Heitbrock, 2007). Besides, lymphoblastoid cells have significant enrichment in rheumatoid arthritis, primary biliary cirrhosis, multiple sclerosis and lupus, concordant with their roles in these diseases (Disanto *et al.*, 2012). For lipids traits—HDL, LDL triglycerides and total cholesterol, liver cells show the most significant enrichment. In addition, lipid traits are enriched in monocytes, consistent with previous findings (Krychtiuk *et al.*, 2014). For psychological diseases/traits including neuroticism, SCZ and years of education, multiple brain tissues are relevant, including angular gyrus, cingulate gyrus, anterior caudate and inferior temporal lobe. Interestingly, body mass index (BMI) has a similar enrichment pattern as SCZ and years of education. Indeed, a recent GWAS result identified 63 shared loci between BMI and SCZ (Bahrami *et al.*, 2020) and earlier study found the inverse association between BMI and education level (Hermann *et al.*, 2011). For SCZ, PALM ranks K562 leukemia cells as an important annotation. Since SCZ is suggested to be linked to immune system (Pantelis *et al.*, 2014), Myint *et al.* (2020) chose K562 cells to examine the regulatory function of SCZ’s associated SNPs and found that more than 10% of SCZ’s associated SNPs show statistically significant allelic difference in driving reporter gene expression in K562 cells. This suggests that SCZ risk SNPs in K562 cells indeed have strong functional annotation signals. Also notice that adipose cells have a close relationship with T2D, in line with the well-known result that the development of T2D involves adipose tissue dysfunction, which links obesity to T2D (Guilherme *et al.*, 2008).

## 4 Conclusion

We proposed a novel statistical method, PALM, to integrate the cell-type/tissue-specific functional annotations with GWAS summary statistics. Comparing with existing methods, PALM can adaptively model the flexible relationship among functional covariates and accommodate a great number of functional annotations. Both simulation studies and real data analysis demonstrate its great power in risk variants prioritization with FDR controlled at the nominal level. Moreover, PALM provides a statistically feasible way to evaluate the relative importance of each covariate, which makes the model more interpretable. From the perspective of computing, the developed EM algorithm is efficient and can scale up to millions of genetic variants and a large number of annotations. We believe that PALM can serve as a useful tool for risk SNP prioritization.

## Supplementary Material

btad068_Supplementary_Data
