# Supplementary material for: PALM: a powerful and adaptive latent model for prioritizing risk variants with functional annotations
Source: Bioinformatics. 2023 Feb 6;39(2):btad068. doi: 10.1093/bioinformatics/btad068 (PMC9950853; doi:10.1093/bioinformatics/btad068)
Supplement: btad068_Supplementary_Data [file btad068_supplementary_data.pdf]

Supporting information for "PALM: A Powerful and Adaptive Latent Model for Prioritizing Risk Variants with Functional Annotations"

Xinyi Yu[1,2], Jiashun Xiao[1,2], Mingxuan Cai[2,3], Yuling Jiao[4], Xiang Wan[1,*], Jin Liu[5,6,*] and Can Yang[2,*]

[1]Shenzhen Research Institute of Big Data, Shenzhen, China
[2]Department of Mathematics, The Hong Kong University of Science and Technology, Hong Kong
[3]Department of Biostatistics, City University of Hong Kong, Hong Kong
[4]School of Mathematics and Statistics, Wuhan University, Wuhan, China
[5]Centre for Quantitative Medicine, Health Services & Systems Research, Duke-NUS Medical School
[6]School of Data Science, The Chinese University of Hong Kong-Shenzhen, Shenzhen, China
[*]To whom correspondence should be addressed.

Contents

1 More simulation results **3**
- 1.1 Simulations when z-values are from other distributions 3
- 1.2 Simulations when z-values are generated from a linear model 7
- 1.3 Quantification of interaction effects between two annotations 9
- 1.4 The influence of missing annotations on the performance of PALM on simulated data 10
- 1.5 The influence of missing annotations on the performance of PALM on real data 14
- 1.6 The influence of cross-validation folds on the performance of PALM 15
- 1.7 The influence of shrinkage parameter on PALM 16
- 1.8 The influence of tree depth on PALM 17
- 1.9 The influence of LD effects on PALM 19

2 More about real data analysis **20**
- 2.1 Discussion on the performance of GPA-Tree 20
- 2.2 Performance of PALM with tree depth greater than 2 24
- 2.3 Numbers of prioritized SNPs in 30 GWASs 25
- 2.4 Genic category of prioritized SNPs by PALM 27

2.5 Analysis on four Schizophrenia GWASs with different sample sizes 28
2.6 Analysis on two Years of Education GWASs with different sample sizes 31
2.7 Sources of 30 GWASs 32

1 More simulation results

1.1 Simulations when z-values are from other distributions

In the main simulation, we generate the z-score of each SNP by $z_j \sim N(\mu_j, 1), j = 1, \ldots, M$, where μ_j follows a *bimodal* distribution. We conduct additional simulations to compare the performances of PALM and other methods under alternative distributions of μ.

Scenario	Distribution
big-normal	$N(0, 4^2)$
near-normal	$\frac{2}{3}N(0, 2^2) + \frac{1}{4}N(0, 3^2) + \frac{1}{12}N(0, 4^2)$
skew	$\frac{1}{2}N(-2, 1^2) + \frac{1}{4}N(-1, 1^2) + \frac{1}{6}N(0, 2^2) + \frac{1}{12}N(1, 4^2)$
spiky	$0.4N(0, 0.5^2) + 0.2N(0, 1^2) + 0.2N(0, 3^2) + 0.2N(0, 5^2)$

Table S1: Alternative distributions of μ.

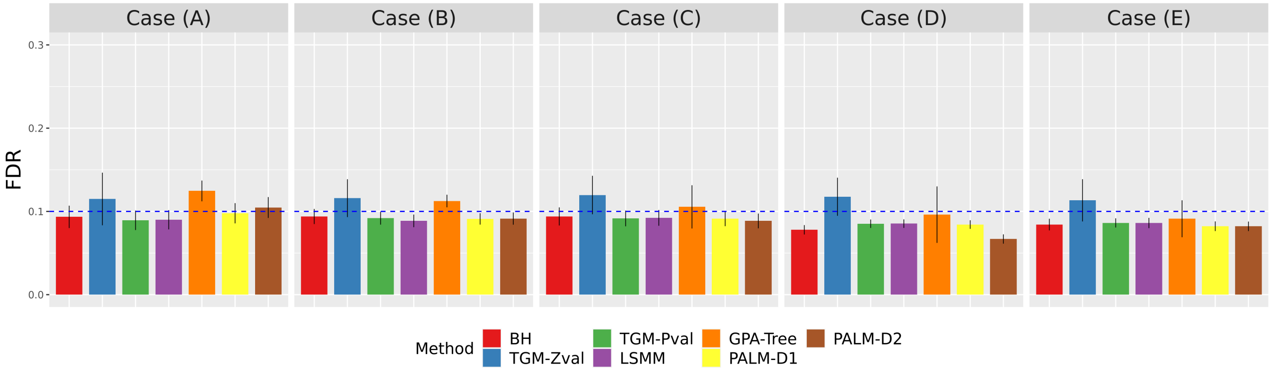

(a) FDR

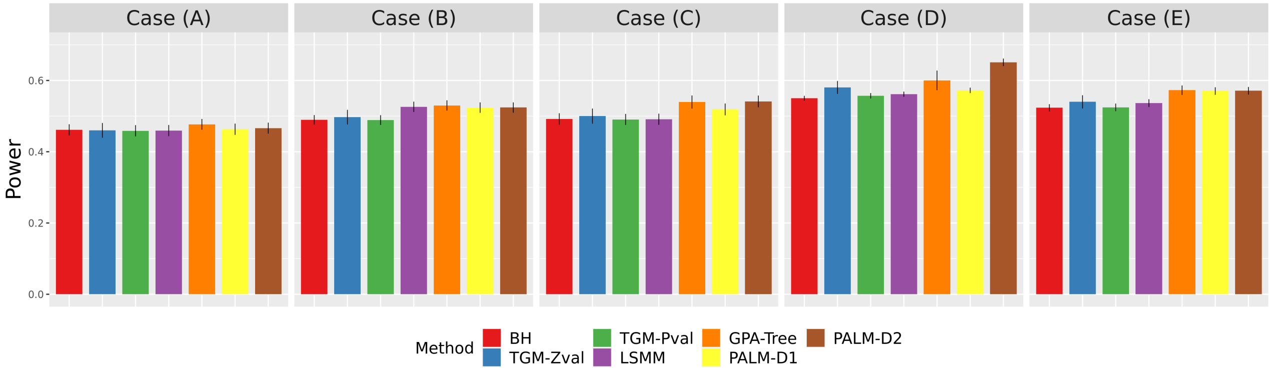

(b) Power

Figure S1: The comparison of PALM-D1 and PALM-D2 with alternative methods, BH, TGM-Pval, TGM-Zval, LSMM and GPA-Tree under $M = 20000$, $D = 50$ and non-null z-score distribution $=$ *big-normal*. The cross-validation fold is $K = 2$ for PALM-D1 and PALM-D2. The results are summarized from 50 replications.

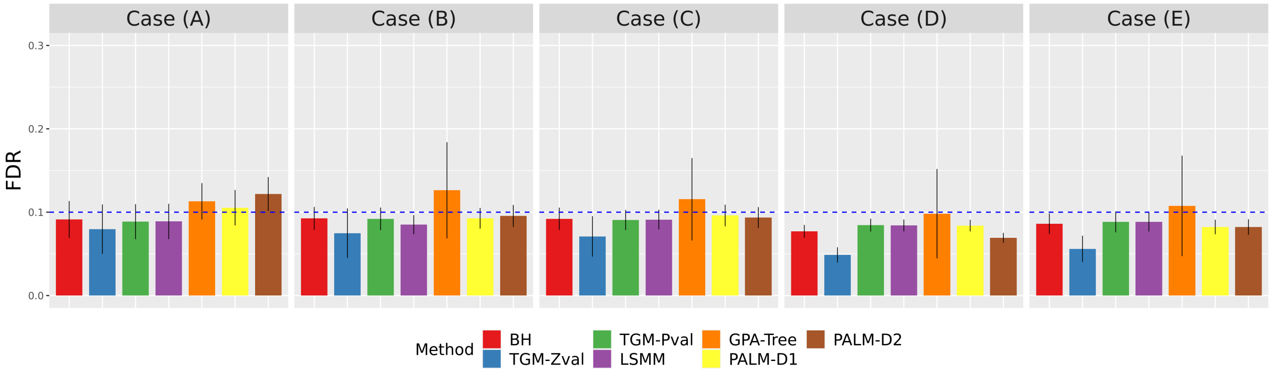

(a) FDR

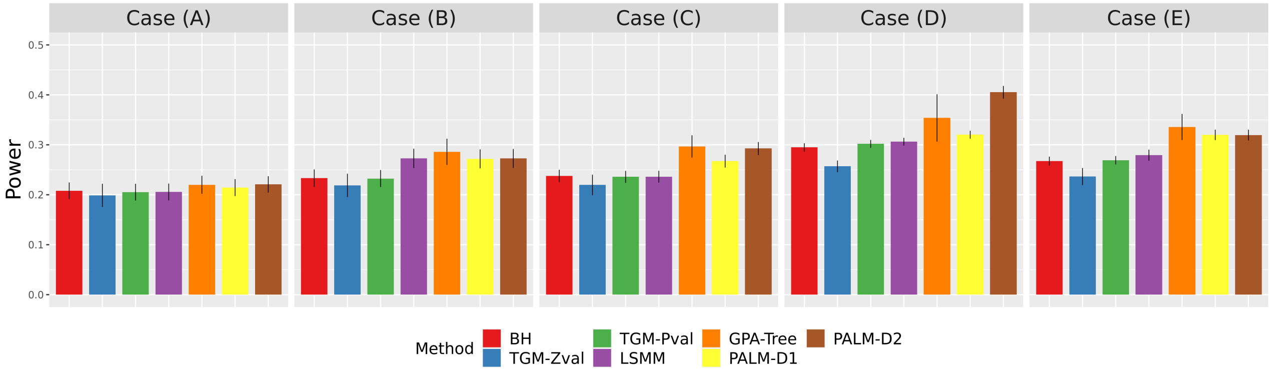

(b) Power

Figure S2: The comparison of PALM-D1 and PALM-D2 with alternative methods, BH, TGM-Pval, TGM-Zval, LSMM and GPA-Tree under $M = 20000$, $D = 50$ and non-null z-score distribution = *near-normal*. The cross-validation fold is $K = 2$ for PALM-D1 and PALM-D2. The results are summarized from 50 replications.

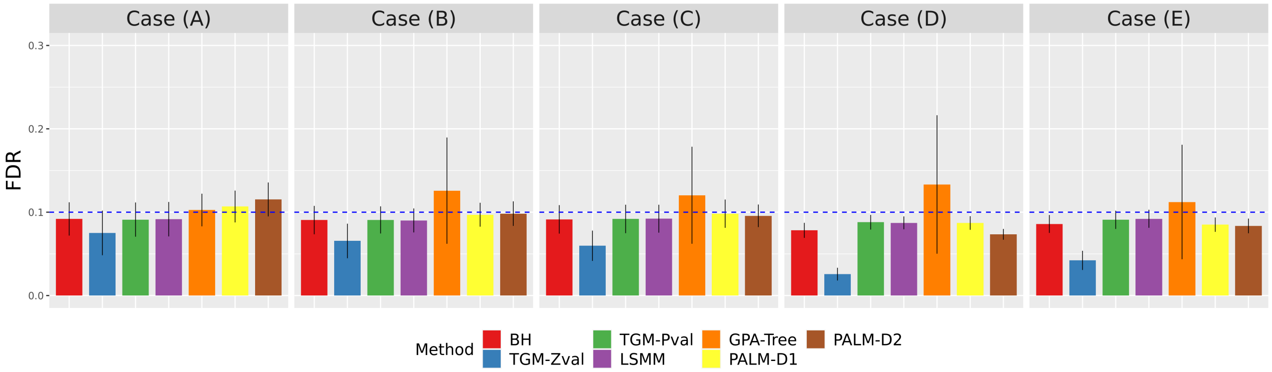

(a) FDR

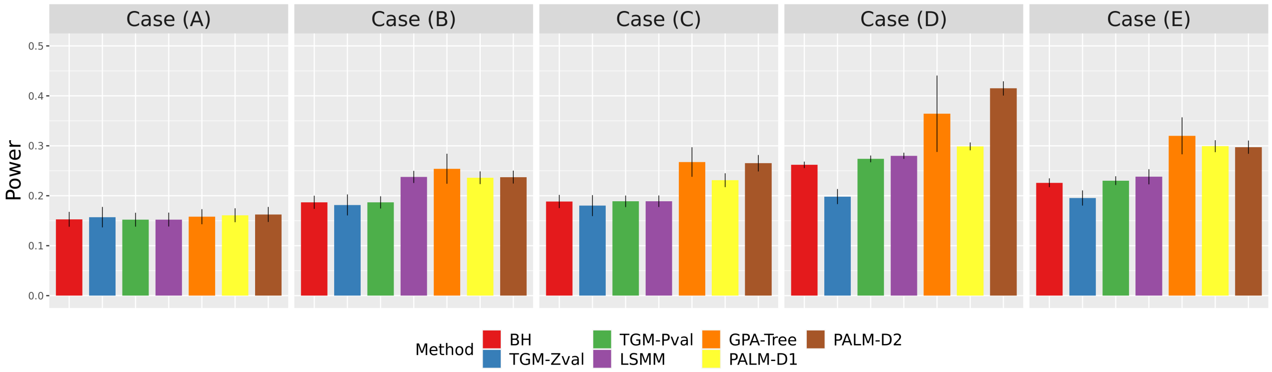

(b) Power

Figure S3: The comparison of PALM-D1 and PALM-D2 with alternative methods, BH, TGM-Pval, TGM-Zval, LSMM and GPA-Tree under $M = 20000$, $D = 50$ and non-null z-score distribution = *skew*. The cross-validation fold is $K = 2$ for PALM-D1 and PALM-D2. The results are summarized from 50 replications.

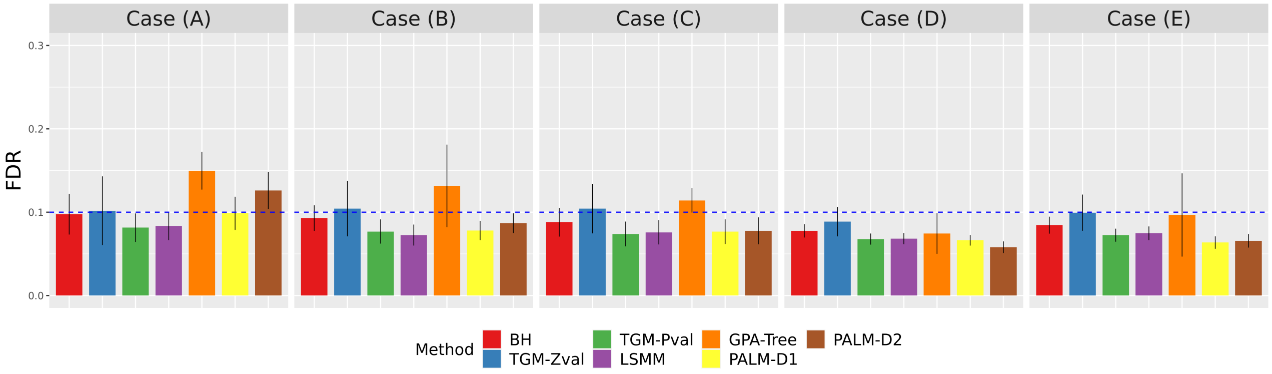

(a) FDR

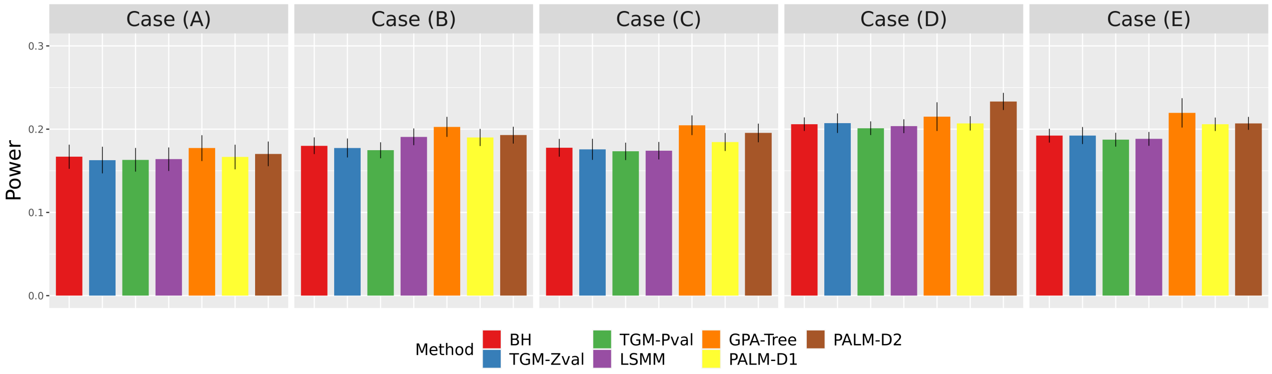

(b) Power

Figure S4: The comparison of PALM-D1 and PALM-D2 with alternative methods, BH, TGM-Pval, TGM-Zval, LSMM and GPA-Tree under $M = 20000$, $D = 50$ and non-null z-score distribution = *spiky*. The cross-validation fold is $K = 2$ for PALM-D1 and PALM-D2. The results are summarized from 50 replications.

1.2 Simulations when z-values are generated from a linear model

In GWAS, the z-scores of SNPs are typically calculated from a linear model with individual data. Specifically, with an assumed heritability h^2 and number of non-null SNPs $N_{nonnull}$, the common linear model takes the form:

$$y = X\beta + \epsilon,$$

where y is the phenotype vector, X is the standardised genotype matrix, $\beta \sim \mathcal{N}(0, \frac{h^2}{N_{nonnull}})$ and $\epsilon \sim \mathcal{N}(0, 1 - h^2)$. The z-score of each SNP can be calculated by performing marginal linear regression. Here we investigate the performance of PALM and compared methods with z-scores generated in this way rather than directly from a prespecified distribution. Since PALM assumes the independence of z-scores and mainly focus on evaluating its performance on the computed z-scores in this section, we will generate genotype matrix with uncorrelated SNPs. For the influence of LD, please refer to supplementary section 1.6.

We consider a realistic setting with heritability $h^2 = 0.2$, sample size $n = 20,000$, total number of SNPs $M = 10,000$ and number of annotation $L = 50$. First, for individual i, we randomly sample M minor allele frequencies (MAFs) from $f_j \sim U[0.05, 0.5], j = 1, ..., M$ and generate genotype g_{ij} of individual i from $g_i \sim Binomial(2, f_j), j = 1, ..., M$ then standardize $\mathbf{g}_j$ to have mean 0 and variance 1 (the standardised genotype is denoted as x_{ij}). Second, following the simulation in the paper, we generate annotation matrix A of shape $M \times L$. Third, the prior probability of each SNP for being in non-null group π_{j1} is calculated by Eq. (9) in the paper and the association status is generated by $Z_j \sim Bernoulli(\pi_{j1}), j = 1, ..., M$. With Z_j, we know the true non-null SNP indices and thus the number of non-null SNPs $N_{nonnull}$. For the null SNPs $j \in \{k|Z_k = 0\}$, their effect sizes are set to be zero; for the non-null SNPs, their effect sizes are randomly sampled by $\beta_j \sim \mathcal{N}(0, \frac{h^2}{N_{nonnull}}), j \in \{k|Z_k = 1\}$. Then the phenotype of each individual i is generated by $y_i = X_i\beta + \epsilon_i, i = 1, ..., n$. Finally, the estimates of effect size of each SNP $\hat{\beta}_j$ and its standard error $se(\hat{\beta}_j)$ can be calculated by regressing y onto X_j. Thus z-score z_j can be obtained by $z_j = \frac{\hat{\beta}_j}{se(\hat{\beta}_j)}$. After the summary statistics have been generated, we can perform and evaluate all the methods just as the simulation in the paper.

Fig. S5 shows the FDR and power for all the methods. Except for GPA-Tree, all the other methods can provide satisfactory FDR control (the following discussion will exclude GPA-Tree). For case (A), the power of methods integrating annotations (LSMM, PALM-D1 and PALM-D2) is of the same level with methods only using summary statistics (BH, TGM-zval and TGM-Pval); for case (B), LSMM, PALM-D1 and PALM-D2 have similarly higher power than methods without using annotations; for case (C)(D)(E), PALM can model nonlinear relationship between annotation and association status thus having significant gain in power compared with other methods. In summary, the results validate the effectiveness of PALM with z-scores generated from a linear model.

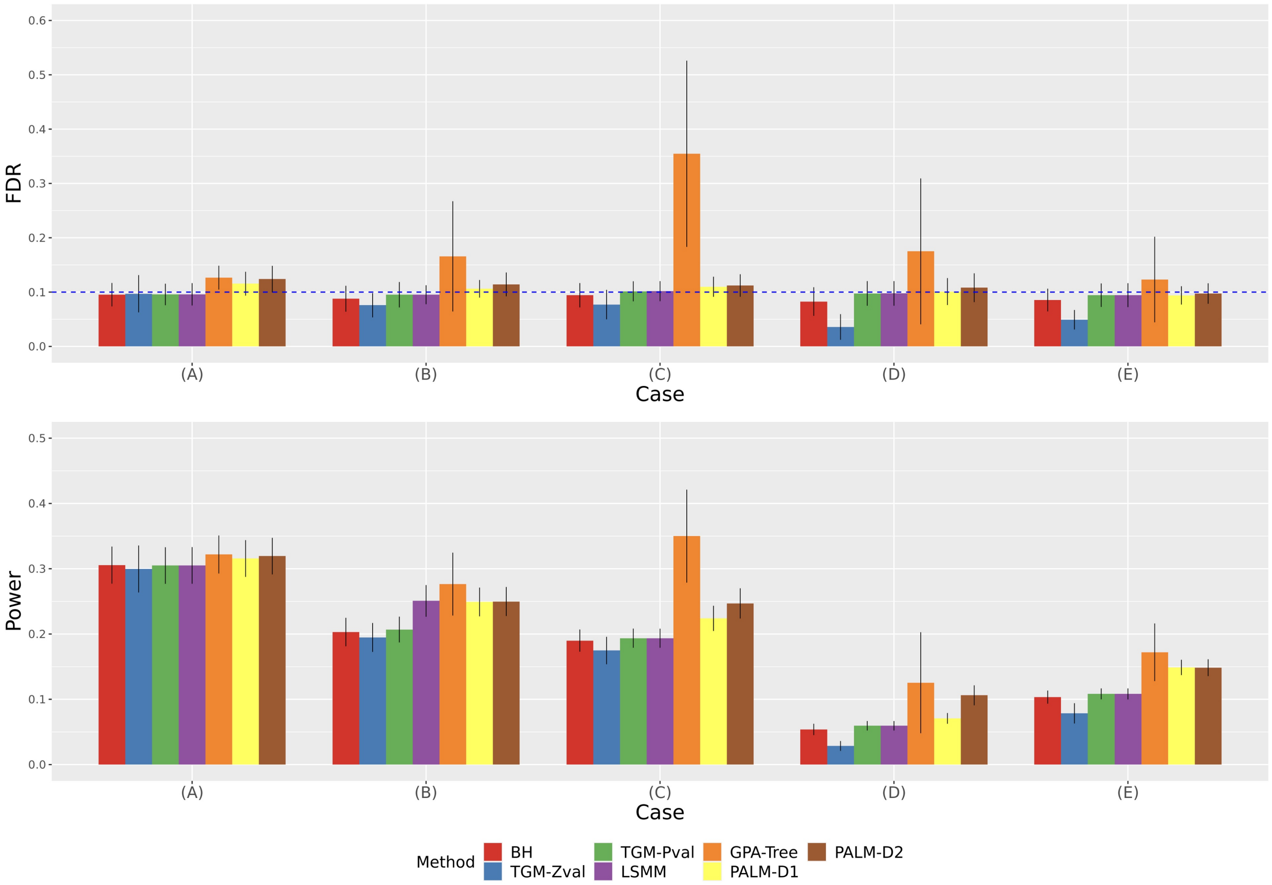

Figure S5: Performance of PALM-D1 and PALM-D2 with other related methods, including BH, TGM-Pval, TGM-Zval, LSMM and GPA-Tree. Heritability $h^2 = 0.2$, number of samples $n = 20,000$, number of SNPs $M = 10,000$, and number of annotations $L = 50$.

1.3 Quantification of interaction effects between two annotations

In this section, we discuss how to quantify the interaction effects between two annotations using PALM. Friedman's H-statistic is introduced to quantify interaction effects [10]. Consider a function F of several features $x_l, l = 1, ..., L$, if two features x_j and x_k don't interact, we can decompose the 2-way partial dependence function [9] in the following way:

$$PD_{jk}(x_j, x_k) = PD_j(x_j) + PD_k(x_k), \tag{1}$$

where $PD_{jk}(x_j, x_k)$ is the 2-way partial dependence function of both features, and $PD_j(x_j)$ and $PD_k(x_k)$ are the partial dependence functions of the single features. If the two features interact, Eq. (1) no longer holds and the difference between the observed 2-way partial dependence and the decomposed one reflects the interaction. Following this idea, the H-statistic measures the interaction between feature x_j and x_k as:

$$H^2_{jk} = \frac{\sum_{i=1}^{n}[PD_{jk}(x_j^{(i)}, x_k^{(i)}) - PD_j(x_j^{(i)}) - PD_k(x_k^{(i)})]^2}{\sum_{i=1}^{n} PD^2_{jk}(x_j^{(i)}, x_k^{(i)})}, \tag{2}$$

where n is the sample size for fitting the model. Theoretically, with centered partial dependence functions, H-statistic is in $[0, 1]$ and a larger H^2_{jk} means a stronger interaction between x_j and x_k. In the context of our paper, the 1-way and 2-way partial dependence functions can be calculated by:

$$PD_j(x_j) = \frac{1}{M}\sum_{i=1}^{M} \hat{F}(x_j, x_{\backslash j}^{(i)}), \tag{3}$$

$$PD_{jk}(x_j, x_k) = \frac{1}{M}\sum_{i=1}^{M} \hat{F}(x_j, x_k, x_{\backslash jk}^{(i)}), \tag{4}$$

where M is the SNP number, $\hat{F}$ is the fitted boosted trees, $\backslash j$ and $\backslash jk$ represent features except j and j, k, respectively.

We implemented the calculation of H-statistic with PALM-D2 (PALM-D1 cannot model interaction). Fig. R1(a) shows the 2-way interactions of the first 5 annotations in case (B), (C), (D) and (E). The H-statistics of feature pairs with interaction effects are significantly larger than zeros while other feature pairs have H-statistics close to zero.

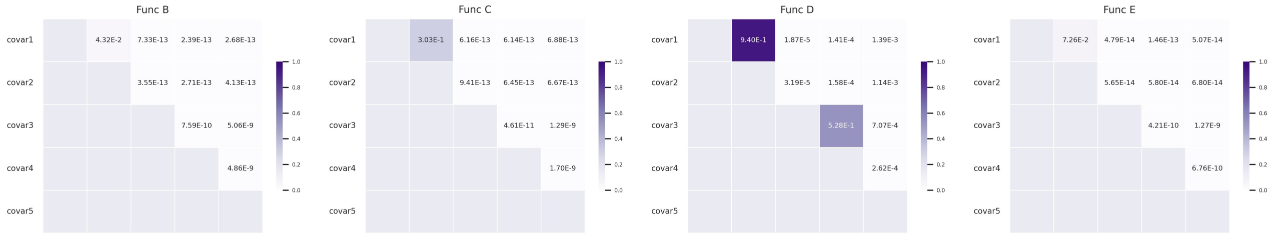

Figure S6: Two-way interactions of the first 5 annotations in case (B), (C), (D) and (E).

1.4 The influence of missing annotations on the performance of PALM on simulated data

As PALM is based on the gradient boosting decision trees, it is capable of handling missing values in functional annotations $\mathbf{A}$. We conduct simulations to gauge the influence of missing value rate of annotation matrix $\mathbf{A}$ on the performance of PALM on simulated data. In details, we consider four values of missing rates, *i.e.*, $mrate \in \{0.05, 0.1, 0.2, 0.4\}$, and use 2-fold cross-validation to select the optimal number of trees. The missing elements in the synthetic annotation matrix $\mathbf{A}$ are randomly chosed.

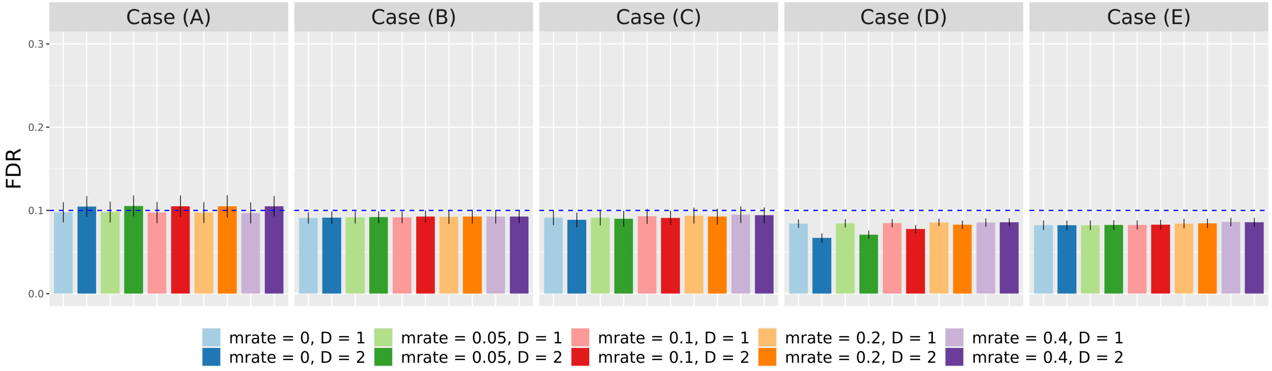

(a) FDR

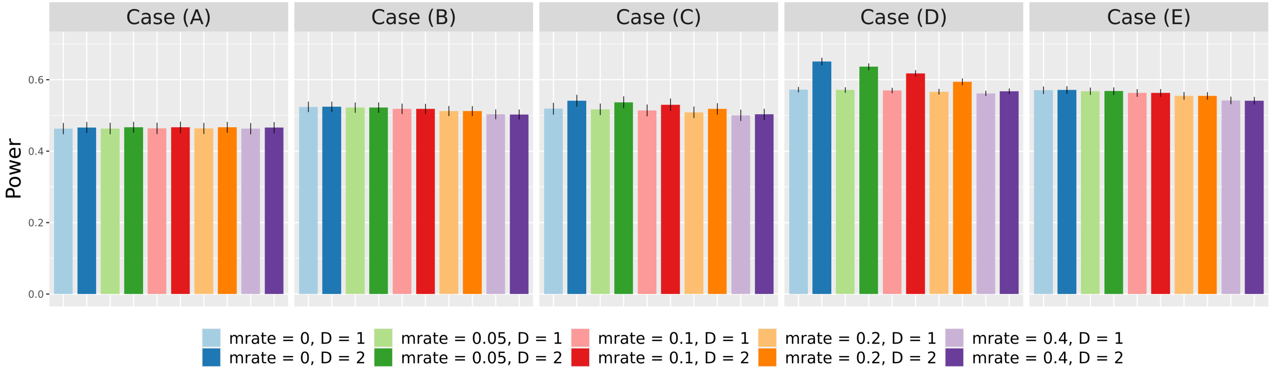

(b) Power

Figure S7: The comparison of PALM-D1 and PALM-D2 under different missing rates. $M = 20000$, $D = 50$ and non-null z-score distribution = *big-normal*. The results are summarized from 50 replications.

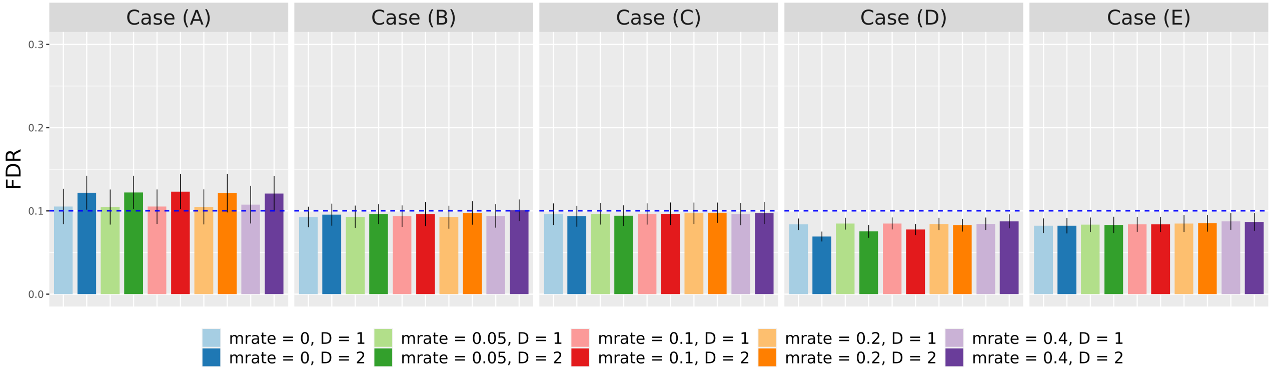

(a) FDR

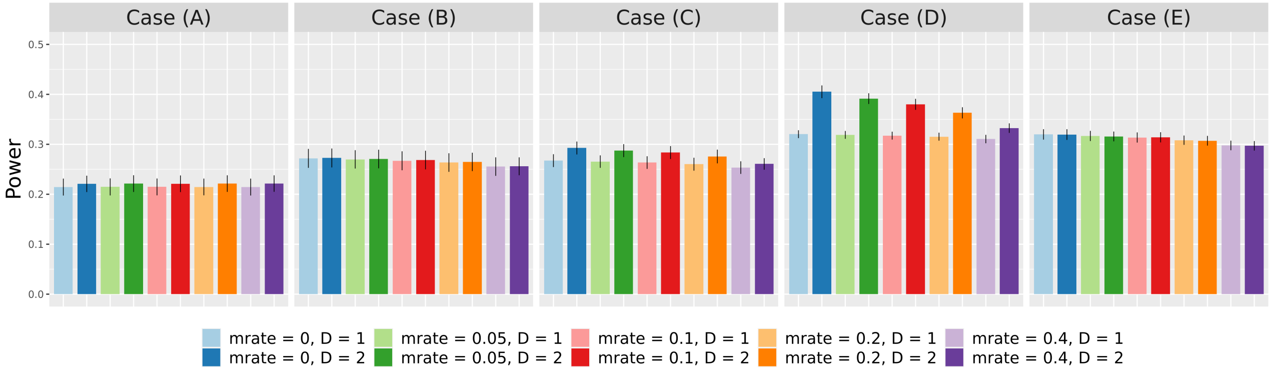

(b) Power

Figure S8: The comparison of PALM-D1 and PALM-D2 under different missing rates. $M = 20000$, $D = 50$ and non-null z-score distribution = *near-normal*. The results are summarized from 50 replications.

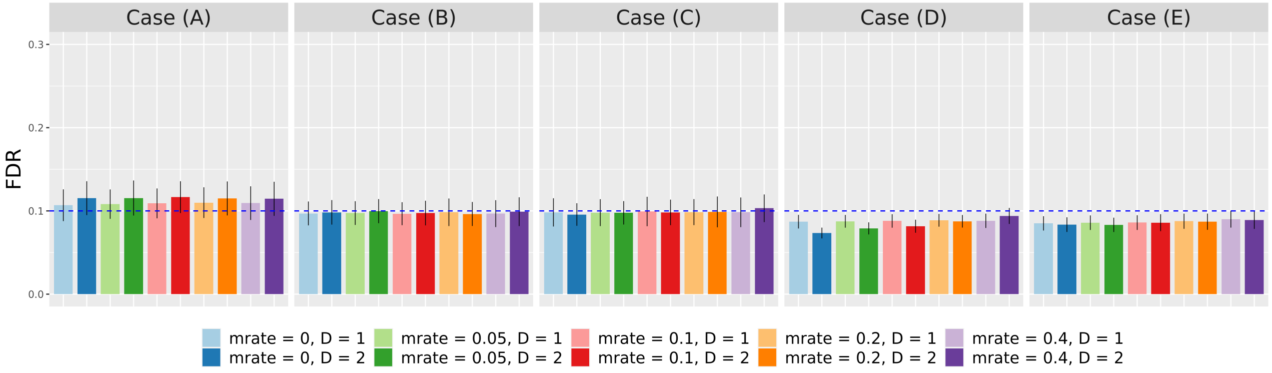

(a) FDR

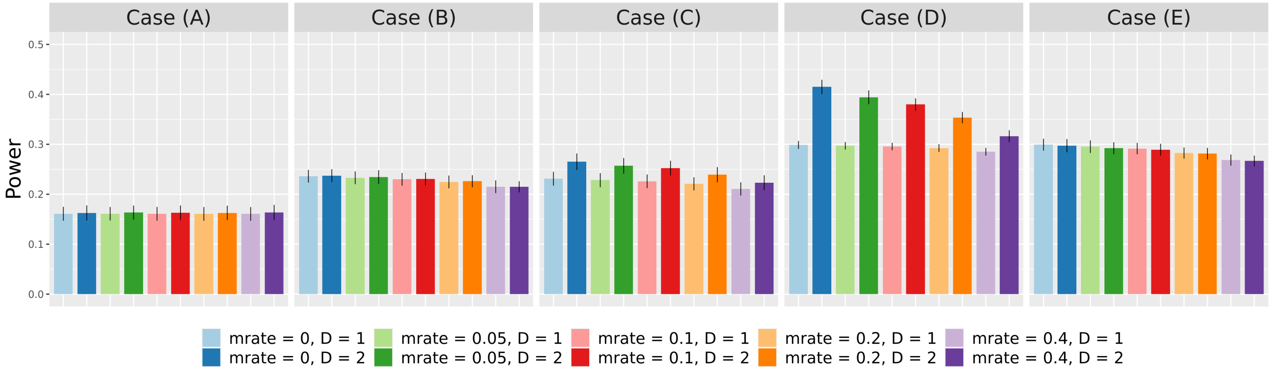

(b) Power

Figure S9: The comparison of PALM-D1 and PALM-D2 under different missing rates. $M = 20000$, $D = 50$ and non-null z-score distribution = *skew*. The results are summarized from 50 replications.

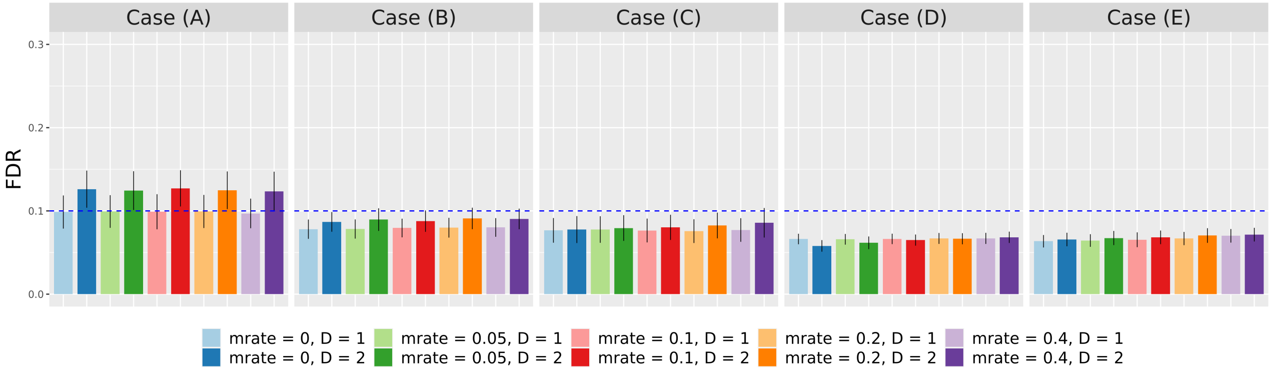

(a) FDR

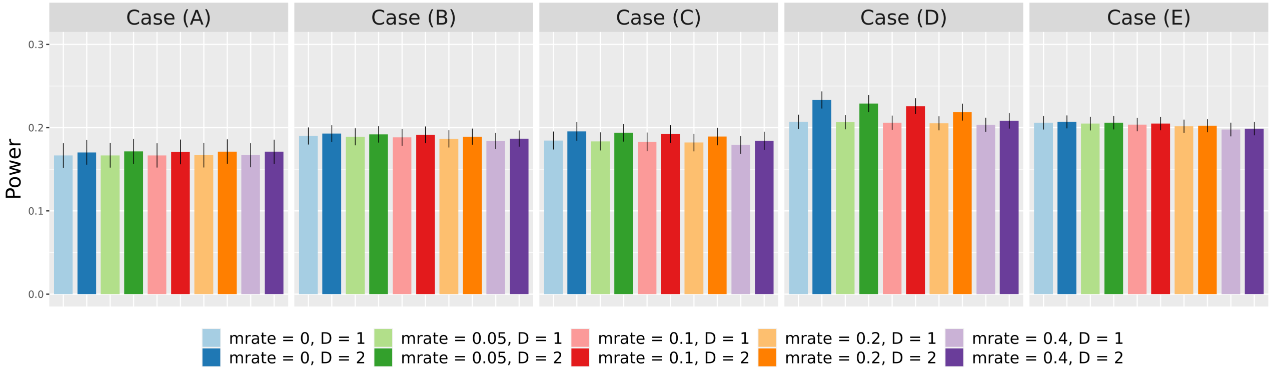

(b) Power

Figure S10: The comparison of PALM-D1 and PALM-D2 under different missing rates. $M = 20000$, $D = 50$ and non-null z-score distribution = *spiky*. The results are summarized from 50 replications.

1.5 The influence of missing annotations on the performance of PALM on real data

We also conduct simulations to gauge the influence of missing value rate of annotation matrix **A** on the performance of PALM on real data. In details, we consider four values of missing rates, *i.e.*, $mrate \in \{0.05, 0.1, 0.2, 0.4\}$, and use 2-fold cross-validation to select the optimal number of trees. The missing elements in the real annotation matrix **A** are randomly chosen. In general, the number of prioritized SNPs will decrease as the missing rate of annotation matrix increases. The two exceptions are Alzheimer's disease and HIV, whose numbers of prioritized SNPs under $mrate = 0.2, 0.4$ are slightly greater than those of without missing values. The figure below therefore excludes these two GWASs for the sake of visualization.

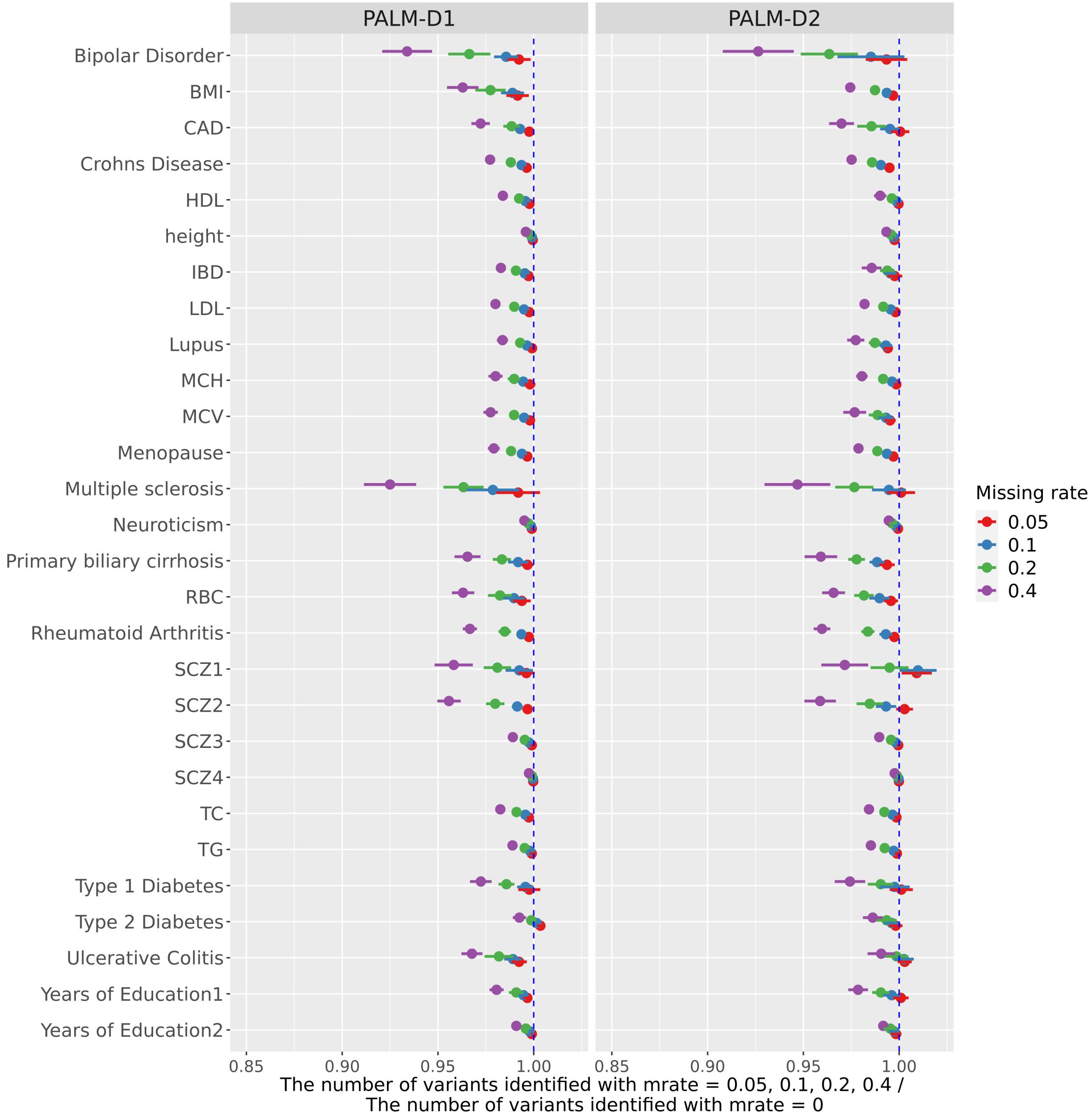

Figure S11: The number of risk variants identified by PALM-D1 and PALM-D2 on real data, under under different missing value rates of annotation matrix. For visualization purpose, these numbers are normalized by dividing the corresponding number of variants identified with $mrate = 0$. The number of variants identified are summarized from 30 replications.

1.6 The influence of cross-validation folds on the performance of PALM

In the main simulation, the optimal number of trees is selected by 5-fold cross validation. We conduct simulations to gauge the influence of cross-validation (CV) folds on the performance of PALM. Specifically, we apply PALM with 2-fold CV and 5-fold CV to the same simulated data then evaluate FDR and power.

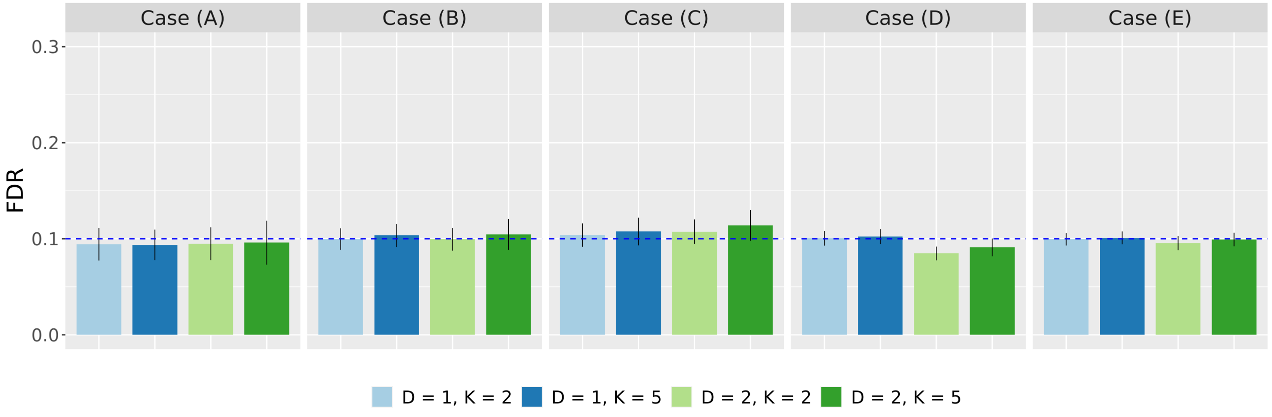

Figure S12: FDR

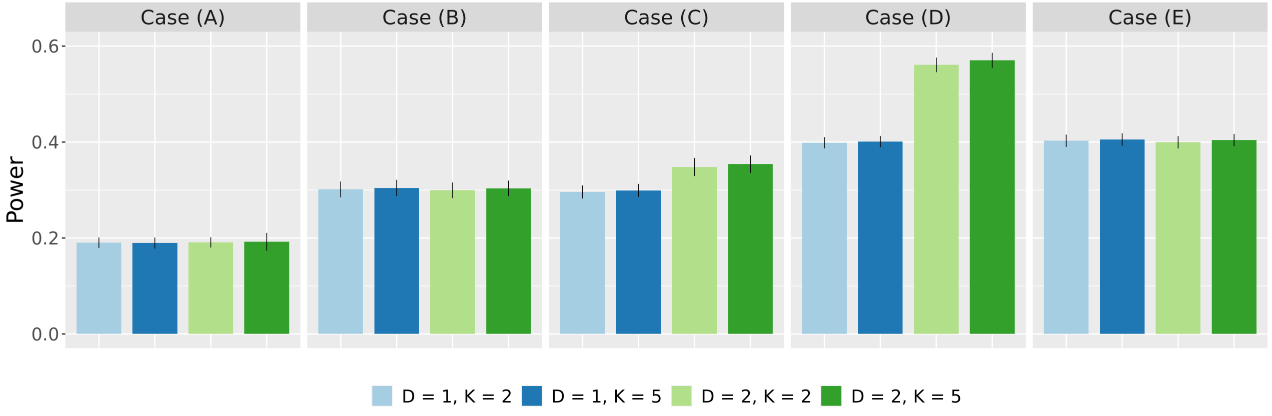

Figure S13: Power

Figure S14: The comparison of PALM-D1 and PALM-D2 with different cross-validation folds ($K \in \{2, 5\}$) under $M = 20000$, $D = 50$ and non-null z-score distribution = *bimodal*. The results are summarized from 30 replications.

1.7 The influence of shrinkage parameter on PALM

In the main paper, we fix the shrinkage parameter ν to be 0.1. Here, we use several different shrinkage parameters $\nu \in \{0.01, 0.05, 0.2, 0.4, 0.8\}$ to run PALM under the same simulation setting as Fig 1(a) with $M = 20,000$ and $L = 50$. From Figure S15(a), we observe that the magnitude of shrinkage parameter has minor impact on FDR control and power: for PALM-D1, the estimated FDR and power with different ν under the five cases are almost the same. For PALM-D2, FDR with $\nu = 0.8$ in case (C) is slightly higher than other ν's but still in the tolerable range; power with $\nu = 0.01$ in case (D) is slightly lower than other ν's. Apart from the two exceptions, the differences on FDR and power between different shrinkage parameters are inapparent. Thus, the performance of PALM is insensitive to the choice of shrinkage parameters, as long as the number of trees is determined by cross-validation.

Although shrinkage parameter shows little influence on risk SNP prioritization, it has some impact on the number of trees of the final model after cross validation. As displayed in Figure S15, with a very small shrinkage parameter (e.g $\nu = 0.01, 0.05$), the number of trees in the final model increases, i.e., the EM algorithm needs more iterations, thus more time-consuming.

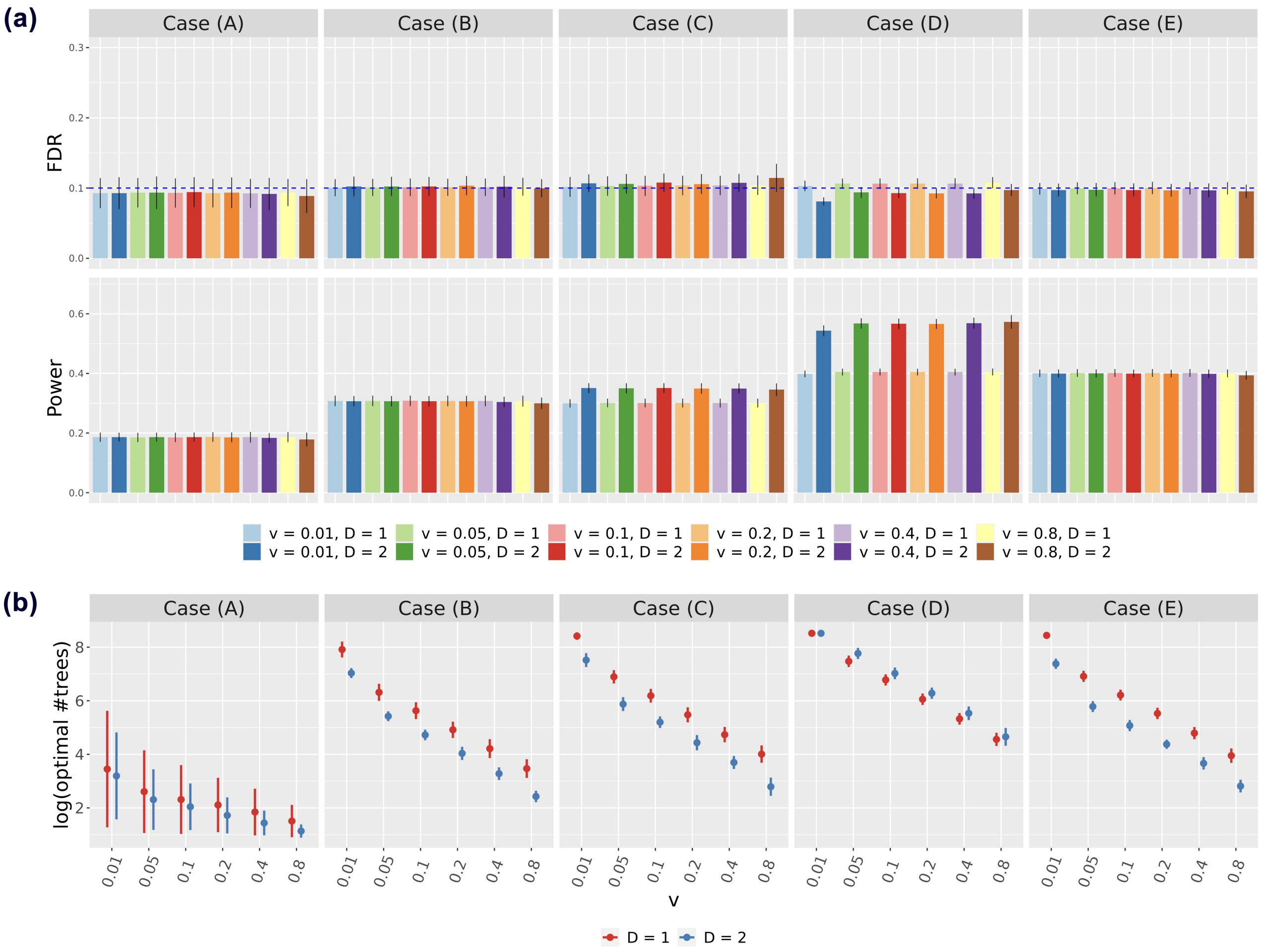

Figure S15: The influence of shrinkage parameter ν on (a) the performances of PALM-D1 and PALM-D2 and (b) the log optimal number of trees of PALM-D1 and PALM-D2. ν and D in the figures represent the shrinkage parameter and tree depth, respectively.

1.8 The influence of tree depth on PALM

We have extensively explored PALM with tree depth 1 and 2. Here, we performance PALM with tree depth 3 and 4 (PALM-D3 and PALM-D4 for short) under the same simulation settings in paper with SNP number $M = 20,000$ and annotation number $L = 50$. Figure S16(a) implies that PALM-D3 can still control FDR while PALM-D4 shows a little inflation on FDR in case (B) and case (C). In terms of statistical power, PALM-D3 and PALM-D4 show no further improvement compared with PALM-D2 in case (C) and case (D). Although the optimal numbers of optimal trees of PALM-D3 and PALM-D4 are smaller than PALM-D1 and PALM-D2, the total computational times of PALM-D3 and PALM-D4 are notably higher since a larger individual tree costs more time to be fitted.

In summary, if we aim to identify more risk SNPs and the computation cost is not a concern, PALM-D2 is recommended; if we want to be more conservative and speed up the prioritization, then PALM-D1 is a great choice. We don't recommend to use trees with depth greater than 2 for risk SNP prioritization.

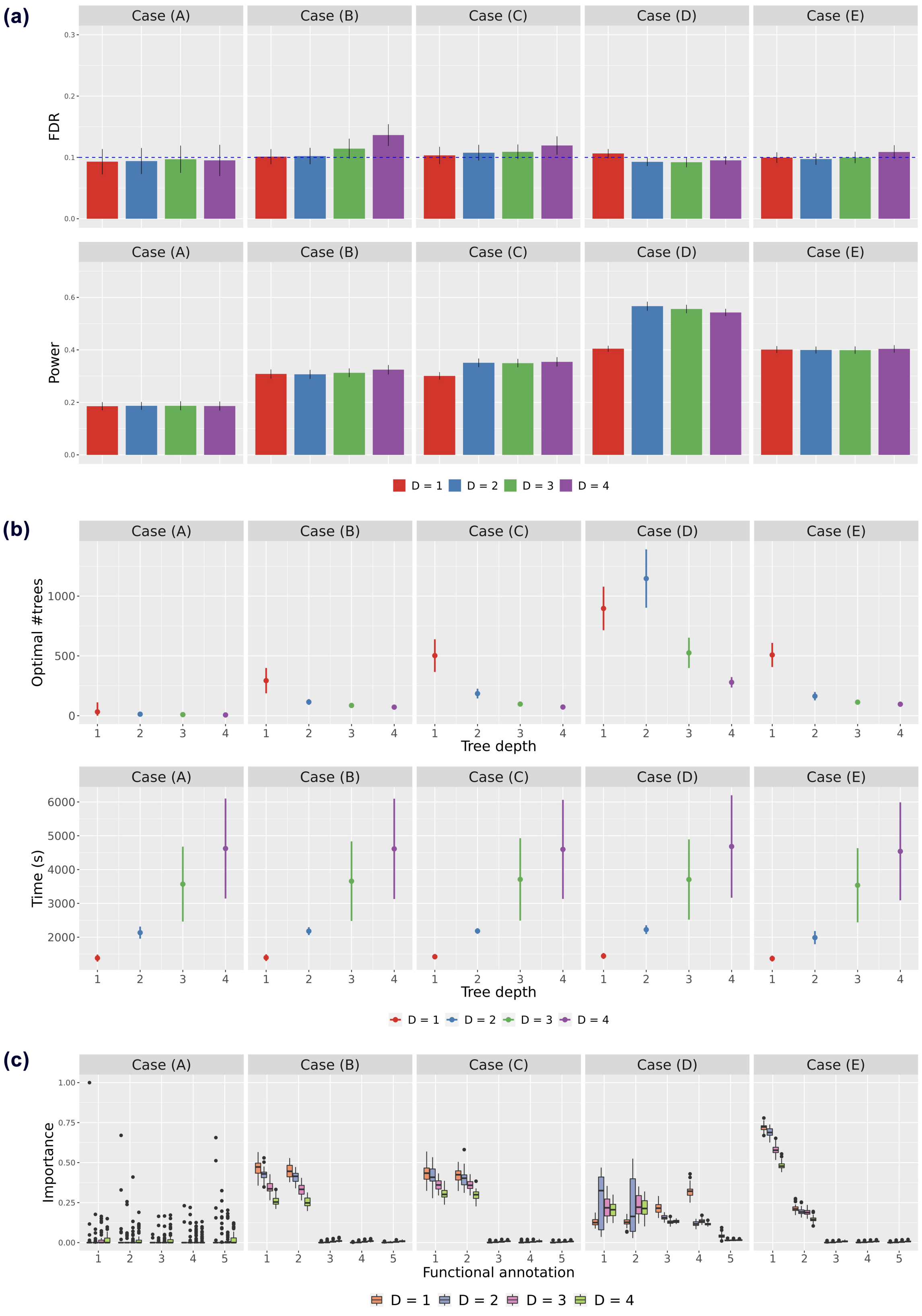

Figure S16: Comparison between PALM-D1, PALM-D2, PALM-D3 and PALM-D4 on (a) FDR and power of risk SNP prioritization, (b) optimal number of trees and total computational time, and (c) evaluated variable importance.

1.9 The influence of LD effects on PALM

Following LSMM, we study the influence of LD effects on PALM using real genotype data from The Wellcome Trust Case Control Consortium (WTCCC). We consider 23170 SNPs in chromosome 1 after quality control and randomly select 23 SNPs as true causal SNPs. We assume the 23 causal SNPs explain 5% phenotypic variance. We use GCTA to simulate phenotypes and PLINK to calculate p-values for all SNPs. We simulate 110 annotations: SNPs within 1Mb of causal SNPs are annotated by the first 10 annotations and by 20 of the last 100 annotations with a probability of 60%. All the other SNPs are annotated with a probability of 10%. We apply two-groups model of p-values (TGM-Pval), PALM-D1 and PALM-D2 to prioritize risk SNPs.

Because of the LD effects, it is unrealistic to detect the true causal SNPs. However, we can expect to identify the regions containing causal SNPs. We set different distance threshold to define the regions around causal SNPs. Any prioritized risk SNP within the regions will be counted as true positive. As shown in Figure S17, without integrating annotations, two-groups model can stably control FDR even under the smallest distance threshold (100kb). Since SNPs within 1Mb of causal SNPs are more likely to be annotated, resulting in the increasing probability of being prioritized, it is difficult for PALM to well control FDR under a small distance threshold. As the distance threshold increases, both PALM-D1 and PALM-D2 become more conservative. We observe that FDR can be controlled at the nominal level 0.1 with a distance threshold of 500kb and 800kb for PALM-D1 and PALM-D2, respectively.

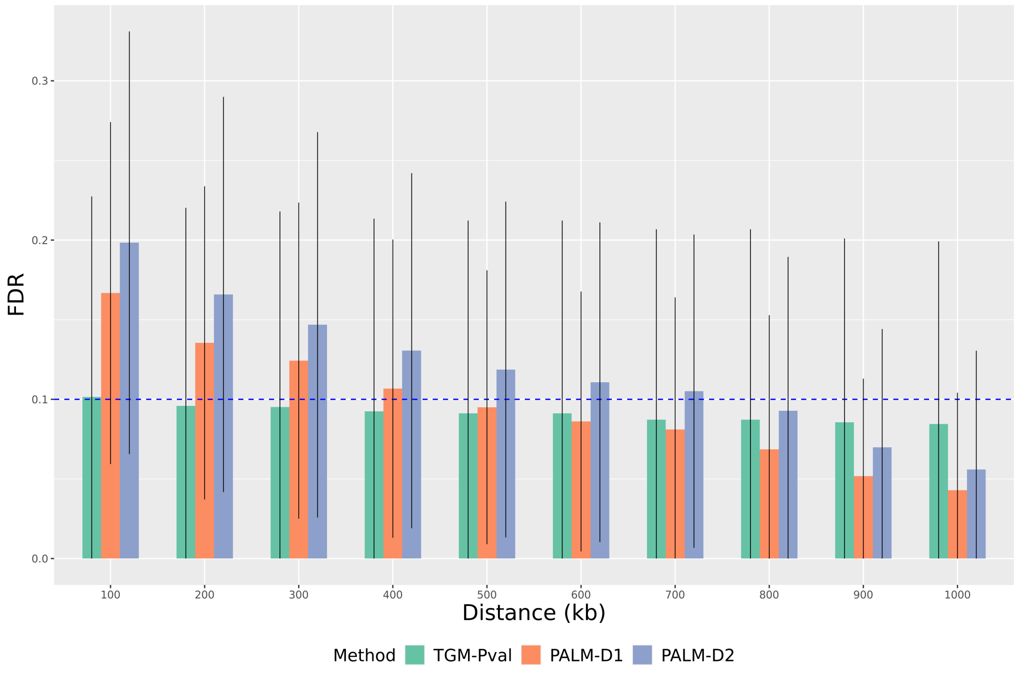

Figure S17: FDR of two-groups model and PALM for risk SNPs prioritization with different distance thresholds. The results are summarized from 50 replications.

2 More about real data analysis

2.1 Discussion on the performance of GPA-Tree

GPA-Tree [12] extends the two-groups model (TGM) by modeling the prior of SNP association status with a single tree. For the algorithm of GPA-Tree, on the first stage, parameter α of the two-groups model is estimated by fitting a linear regression in the M step; on the second stage, with α fixed at its estimation, a full tree is fitted then pruned with a complexity parameter cp in each iteration until the incomplete likelihood no longer increases even with other cp choices. In the simulation study, GPA-Tree did not well control FDR under several cases. In the real data analysis, GPA-Tree did not provide a satisfactory prioritization of risk SNPs: for quite a few traits, it identified even fewer SNPs than TGM; but it identified much more SNPs than other related methods on several traits. It seems that GPA-tree suffers from a stability issue.

To figure out the problem with GPA-Tree, we investigate the model fitting process of GPA-Tree on real data. Figure S18 shows the incomplete log-likelihoods on the first and second stage obtained by GPA-Tree. We observe that for more than half of the GWASs, the log-likelihood at the end of second stage is even smaller than that at the end of first stage; while for several traits, namely HIV, lupus and rheumatoid arthritis, the log-likelihood has a jump on the second stage (these three traits are exactly the ones with much more SNPs prioritized by GPA-Tree). The cause of these phenomena can be attributed to the algorithm design and limitation of the model. In order to prevent overfitting, GPA-Tree pruned the fitted full tree with the complexity parameter cp. An aggressive cp might leads to uncontrolled FDR while a conservative cp can limit the power of risk SNP prioritization. With a common cp (e.g 0.005), it is hard to achieve both good power and FDR control for all GWASs. Hence, for some traits, the log-likelihood using the pruned tree increases compared with that using a linear regression model; but for many other traits, the log-likelihood degrades on the second stage, indicating that the use of pruned tree is not more effective than a linear model; for several other traits, because another smaller cp in the set of candidate cp's has been found, the newly pruned tree is dramatically enlarged, resulting in a sudden increase in log-likelihood. Since there is no stable regularization in GPA-Tree, it cannot credibly and consistently improve risk SNP prioritization.

Besides the instability, we also notice that, for almost all traits without a jump in log-likelihood, the tree structure does not change throughout the second stage of GPA-Tree. Figure S19 shows the tree depths along the iterations on the second stage. Except for HIV, lupus and rheumatoid arthritis, the tree depths of all the other traits remain unchanged during EM iterations. Indeed, not only the tree depths keep the same but also the split nodes barely change throughout the second stage. For instance, the tree structure of BMI only changed once during EM iterations and the final tree only involves two annotations; the tree structure of type 1 diabetes keeps unchanged in all iterations (Figure S20(a)(b)). In other words, only the leaf values are updated in most of the iterations but at the cost of fitting a huge full size tree and pruning, suggesting the low efficiency of GPA-Tree's model fitting process.

We also plot the trees fitted by GPA-Tree of traits with abnormally large number of prioritized SNPs (Figure S20(c)(d)(e) for rheumatoid arthritis, HIV and lupus) and two traits with prioritized SNPs fewer than TGM (Figure S20(f)(g) for multiple sclerosis and

type 2 diabetes). Notice that the annotations used to split nodes in rheumatoid arthritis, HIV and lupus before the jump are in accordance with the important annotations ranked by PALM. Because of the unstable algorithm, however, GPA-Tree fits much larger trees in the following iterations and causes inflation. For multiple sclerosis and type 2 diabetes, although the complexity of the final trees is controlled, it does not fully make use of the annotations. Compared with PALM, the tree of multiple sclerosis does not use the annotation *primary B cells from peripheral blood* which is evaluated to have nonnegligible importance; the tree of type 2 diabetes does not include multiple useful annotations such as *monocytes-CD14+ RO01746 primary cells*, *K562 leukemia cells* and *fetal hearts*. On the contrary, PALM uses a shallow tree to fit the residual in each EM iteration and ensemble these trees. Therefore, each individual tree in PALM can contribute to fitting a complex function in different aspects by choosing several annotations, and the aggregation of trees leads to a strong and stable model.

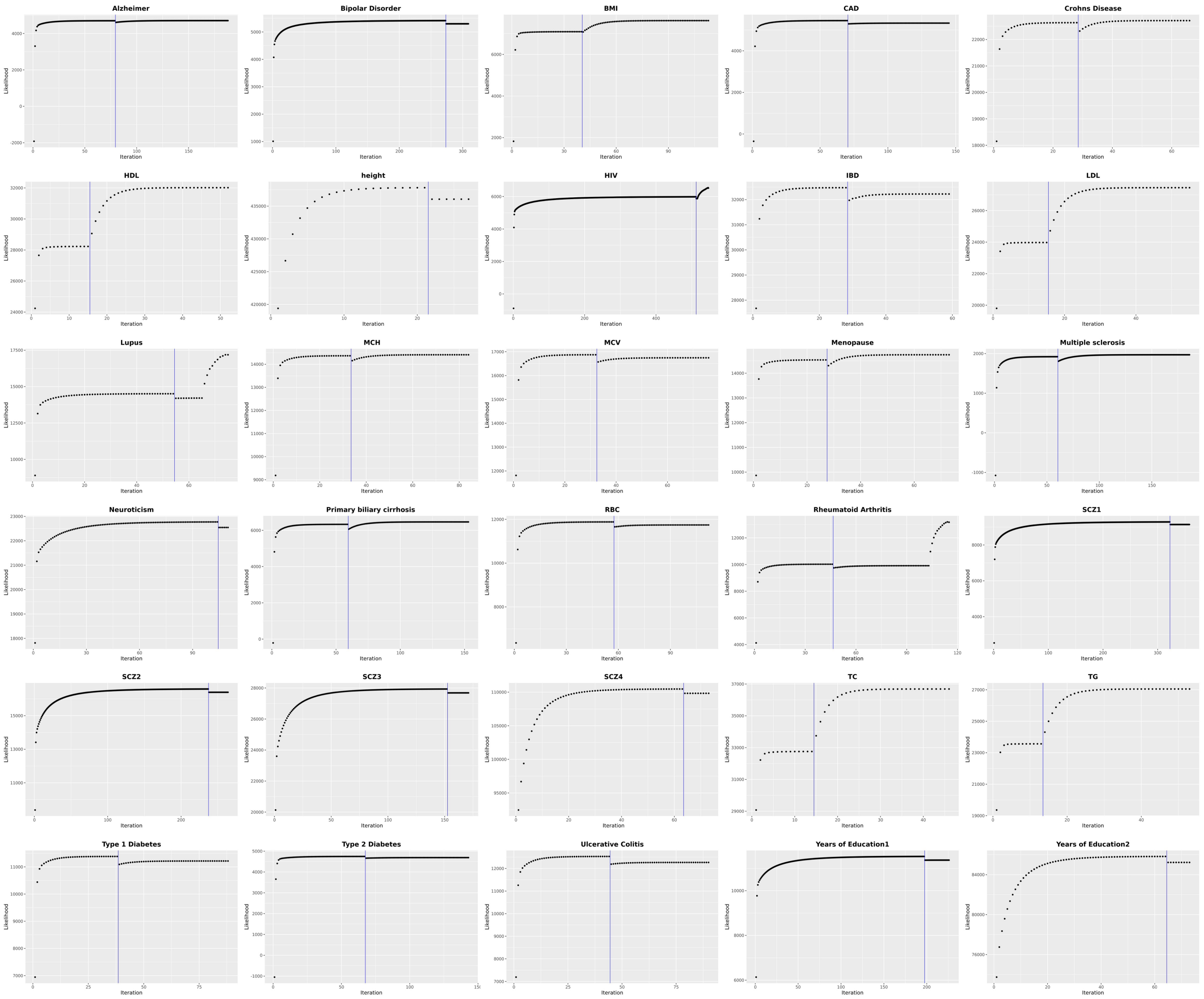

Figure S18: Incomplete log-likelihoods of 30 GWASs by GPA-Tree. Dots on the left and right side of the blue vertical line belongs to the first and second stage of GPA-Tree algorithm, respectively. The complexity parameter $cp = 0.005$.

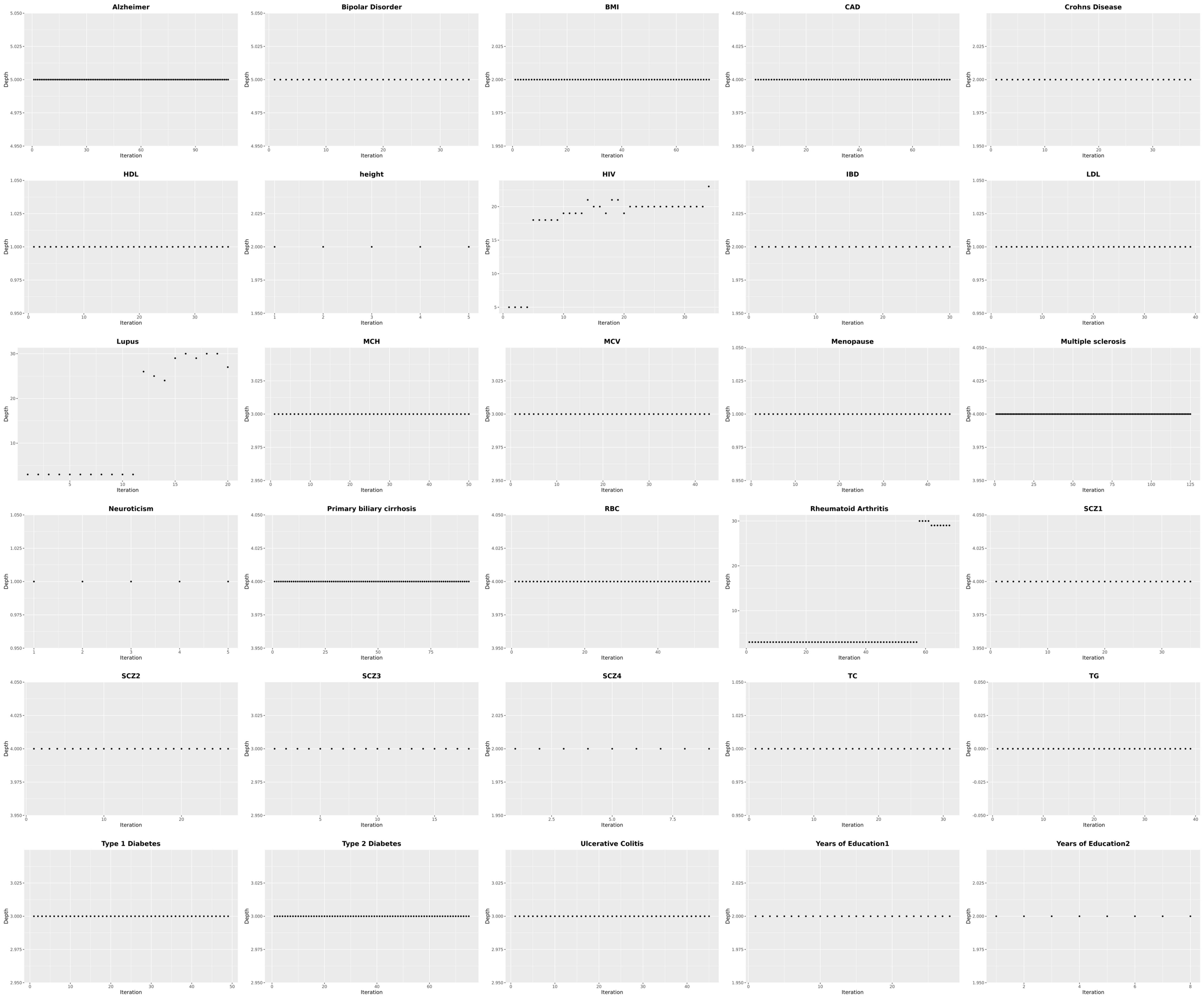

Figure S19: Tree depths of 30 GWASs along the iterations on the second stage of GPA-Tree. The complexity parameter $cp = 0.005$.

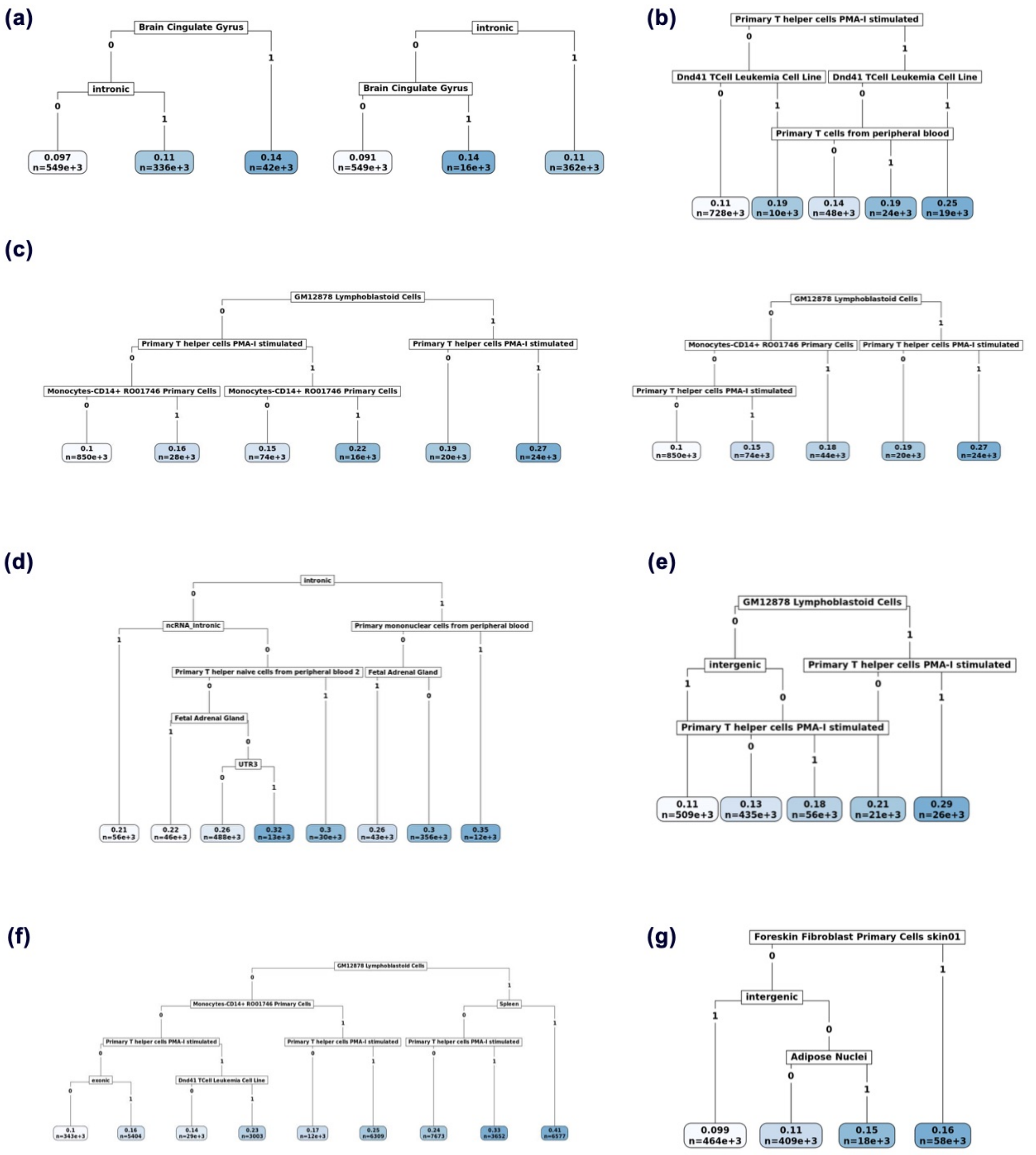

Figure S20: Example fitted trees by GPA-Tree. (a) BMI: tree structure in the 1st and 2nd iterations (left), tree structure in the rest of iterations (right). (b) Type 1 diabetes: pruned tree structure in all iterations. (c) Rheumatoid arthritis: tree structure in the 1st and 2nd iterations (left), tree structure in the 3nd-57th iteration (right). (d) HIV: tree structure in the 1st-4th iterations. (e) Lupus: tree structure in the 11th iteration. Tree structures in the rest iterations of rheumatoid arthritis, HIV and lupus are not shown here due to their large size. (f) Multiple sclerosis: tree structure in all iterations. (g) Type 2 diabetes: tree structure in all iterations.

2.2 Performance of PALM with tree depth greater than 2

Continuing the discussion on tree depth in section 1.8, we perform PALM-D3 and PALM-D4 on real data and add the results to Figure 2 as shwon in Figure S21. The performance of PALM-D3 and PALM-D4 on real data verify the simulation result in S16: with the increase of tree depth, more SNPs can often be identified but PALM-D3 and PALM-D4 may not provide satisfactory FDR control.

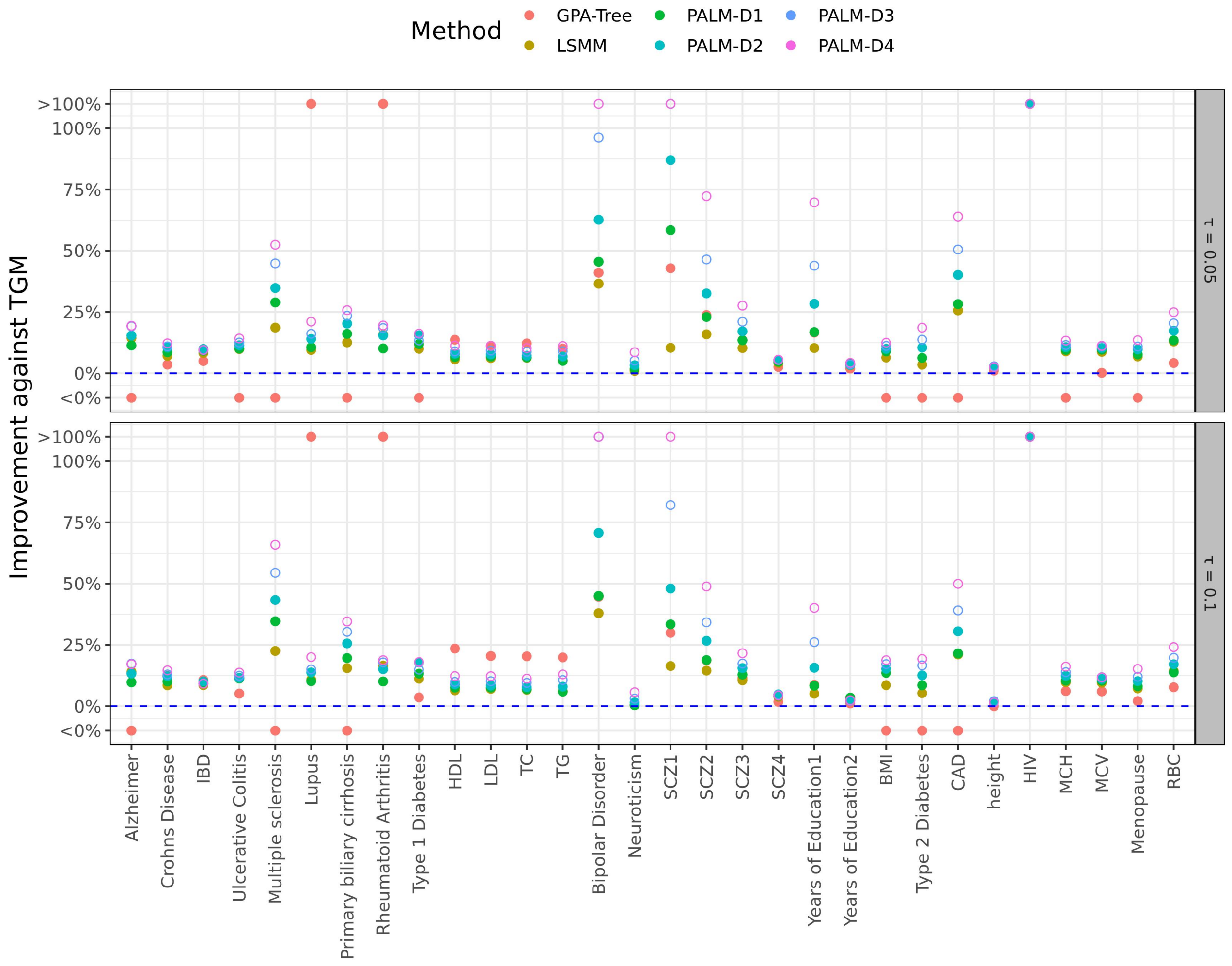

Figure S21: The improvement on the number of prioritized risk SNPs for LSMM, GPA-Tree, PALM-D1, PALM-D2, PALM-D3 and PALM-D4 compared with TGM under the global FDR threshold $\tau = 0.05$ and $\tau = 0.1$.

2.3 Numbers of prioritized SNPs in 30 GWASs

GWAS	TGM	LSMM	GPA-Tree	PALM-D1	PALM-D2
Alzheimer	501	573	436	558	578
Bipolar Disorder	134	183	189	195	218
BMI	1299	1382	1024	1415	1428
CAD	386	485	379	495	541
Crohns Disease	3568	3821	3694	3874	3943
HDL	3108	3284	3533	3313	3355
height	78514	80628	79362	80394	80650
HIV	2	19	1173	7	13
IBD	4928	5335	5173	5382	5416
LDL	2860	3037	3180	3055	3080
Lupus	1726	1890	4161	1909	1968
MCH	2058	2242	2027	2254	2277
MCV	2459	2674	2463	2696	2712
Menopause	2788	2978	2654	3002	3059
Multiple sclerosis	408	484	323	526	550
Neuroticism	3092	3122	3134	3148	3193
Primary biliary cirrhosis	1312	1477	1033	1523	1578
RBC	1436	1622	1496	1629	1685
Rheumatoid Arthritis	1235	1430	4213	1360	1426
SCZ1	77	85	110	122	144
SCZ2	635	736	786	781	842
SCZ3	2800	3088	3180	3177	3279
SCZ4	27446	28590	28151	28735	28920
TC	3984	4235	4469	4241	4270
TG	2423	2548	2667	2545	2588
Type 1 Diabetes	1482	1630	1427	1658	1712
Type 2 Diabetes	430	445	376	457	475
Ulcerative Colitis	1983	2179	1964	2183	2209
Years of Education1	476	525	555	556	611
Years of Education2	18678	19286	19040	19389	19306

Table S2: Numbers of prioritized SNPs of 30 GWASs with different methods under the global FDR threshold $\tau = 0.05$.

GWAS	TGM	LSMM	GPA-Tree	PALM-D1	PALM-D2
Alzheimer	813	927	736	892	921
Bipolar Disorder	311	429	450	451	531
BMI	1918	2082	1659	2178	2210
CAD	819	992	817	995	1069
Crohns Disease	4760	5165	5349	5239	5341
HDL	3638	3872	4493	3913	3961
height	107647	109652	107658	109309	109791
HIV	43	147	2193	109	126
IBD	6905	7496	7644	7549	7602
LDL	3343	3579	4027	3596	3624
Lupus	2882	3194	6952	3175	3279
MCH	2847	3125	3022	3144	3199
MCV	3503	3838	3713	3867	3892
Menopause	3663	3927	3739	3960	4037
Multiple sclerosis	586	718	575	789	840
Neuroticism	5841	5864	5870	5865	5929
Primary biliary cirrhosis	1824	2107	1687	2182	2291
RBC	2378	2718	2561	2706	2784
Rheumatoid Arthritis	2045	2383	7030	2251	2354
SCZ1	458	533	595	611	678
SCZ2	2089	2392	2479	2482	2646
SCZ3	6773	7487	7554	7645	7824
SCZ4	48307	50158	49152	50336	50624
TC	4675	4987	5626	4999	5037
TG	2835	3007	3399	3002	3060
Type 1 Diabetes	2187	2432	2265	2477	2577
Type 2 Diabetes	716	754	645	777	806
Ulcerative Colitis	3030	3377	3185	3372	3381
Years of Education1	1512	1589	1644	1638	1749
Years of Education2	33526	34490	33904	34685	34306

Table S3: Numbers of prioritized SNPs of 30 GWASs with different methods under the global FDR threshold $\tau = 0.1$.

2.4 Genic category of prioritized SNPs by PALM

We use ANNOVAR [26] to obtain the genic categories of SNPs prioritized by PALM. We found that most prioritized SNPs are indeed in the non-coding regions (Figure S22), consistent with the fact that most GWAS hits are in the non-coding regions.

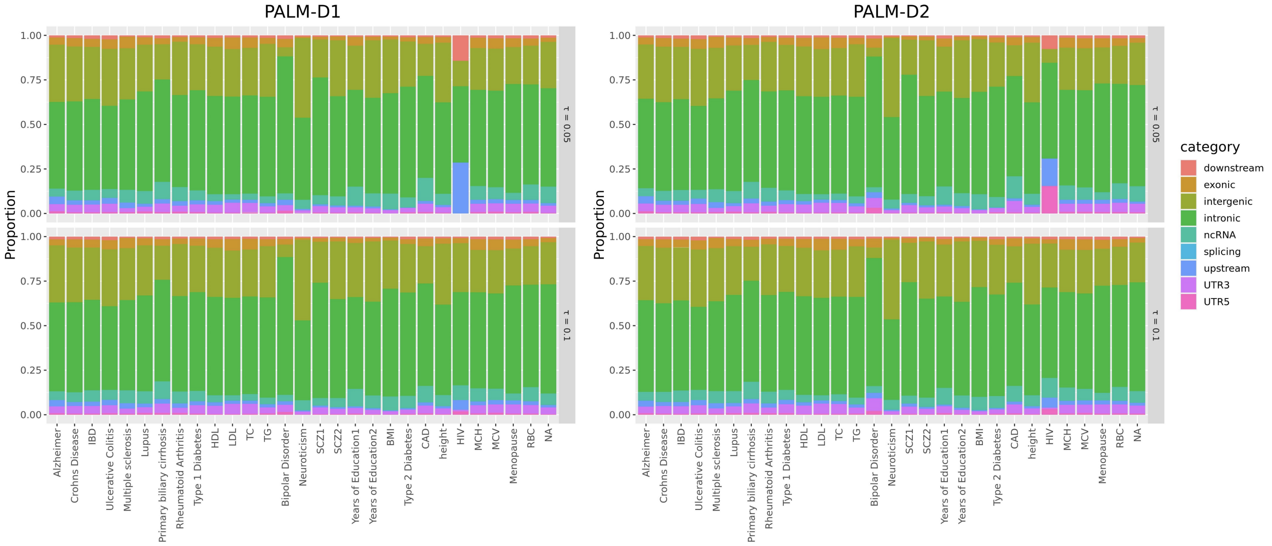

Figure S22: The proportion of 9 genic functional categories of SNPs prioritized by PALM-D1 and PALM-D2 under the FDR threshold $\tau = 0.05$ and $\tau = 0.1$.

2.5 Analysis on four Schizophrenia GWASs with different sample sizes

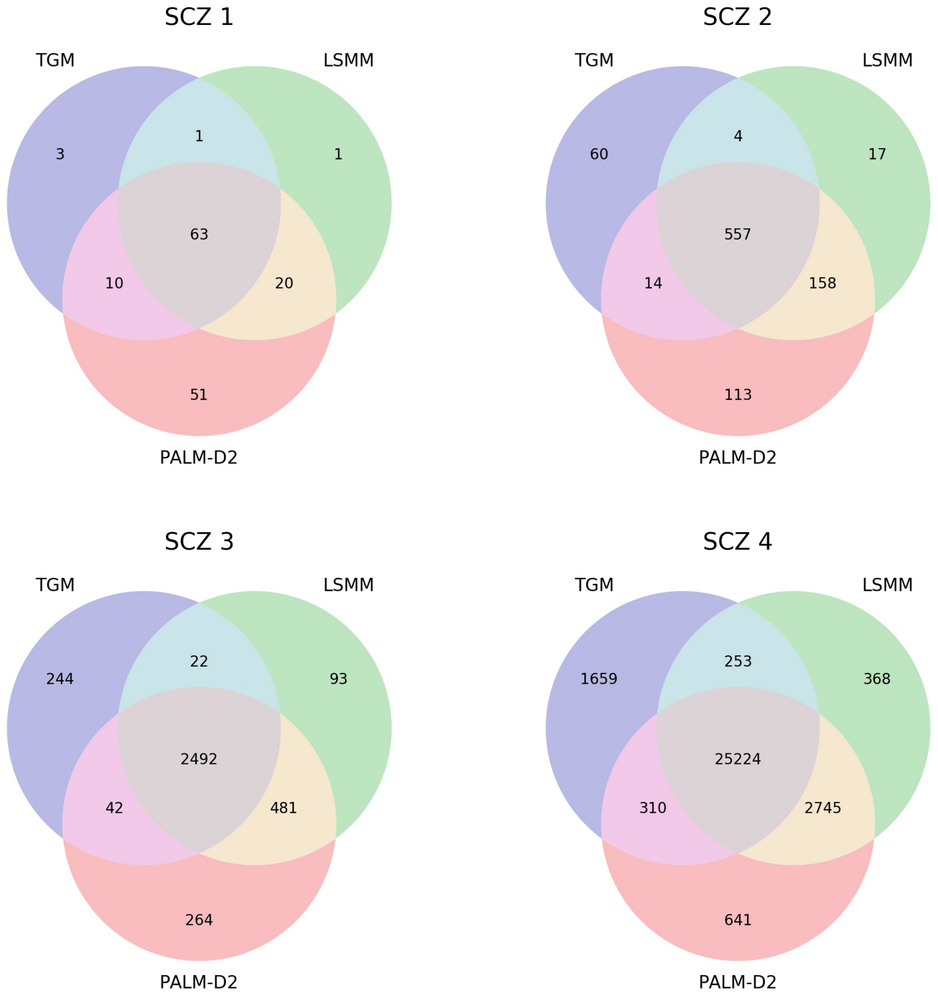

Figure S23: Venn diagrams comparing SNPs prioritized by TGM, LSMM and PALM-D2 of four SCZ GWASs under the FDR control threshold $\tau = 0.05$.

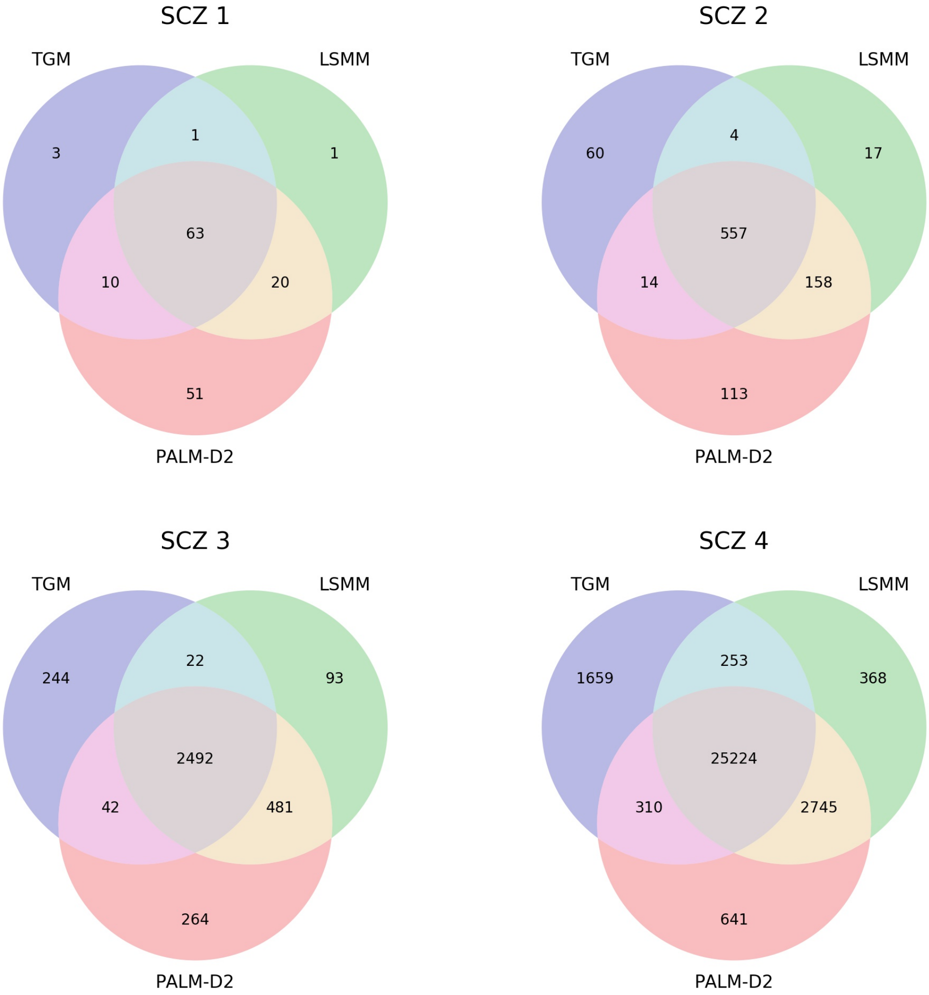

Figure S24: Venn diagrams comparing SNPs prioritized by TGM, LSMM and PALM-D2 of four SCZ GWASs under the FDR control threshold $\tau = 0.1$.

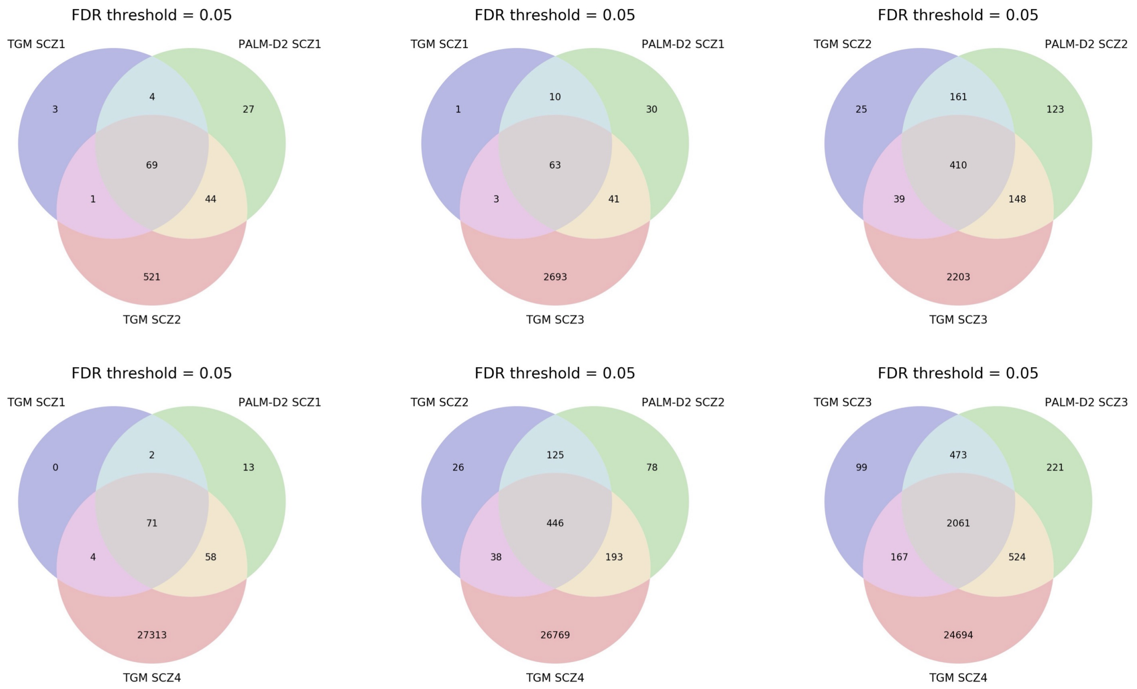

Figure S25: Venn diagrams comparing SNPs prioritized by TGM, PALM-D2 of SCZ GWAS with a smaller sample size and TGM of SCZ GWAS with a larger sample size under the FDR control threshold $\tau = 0.05$.

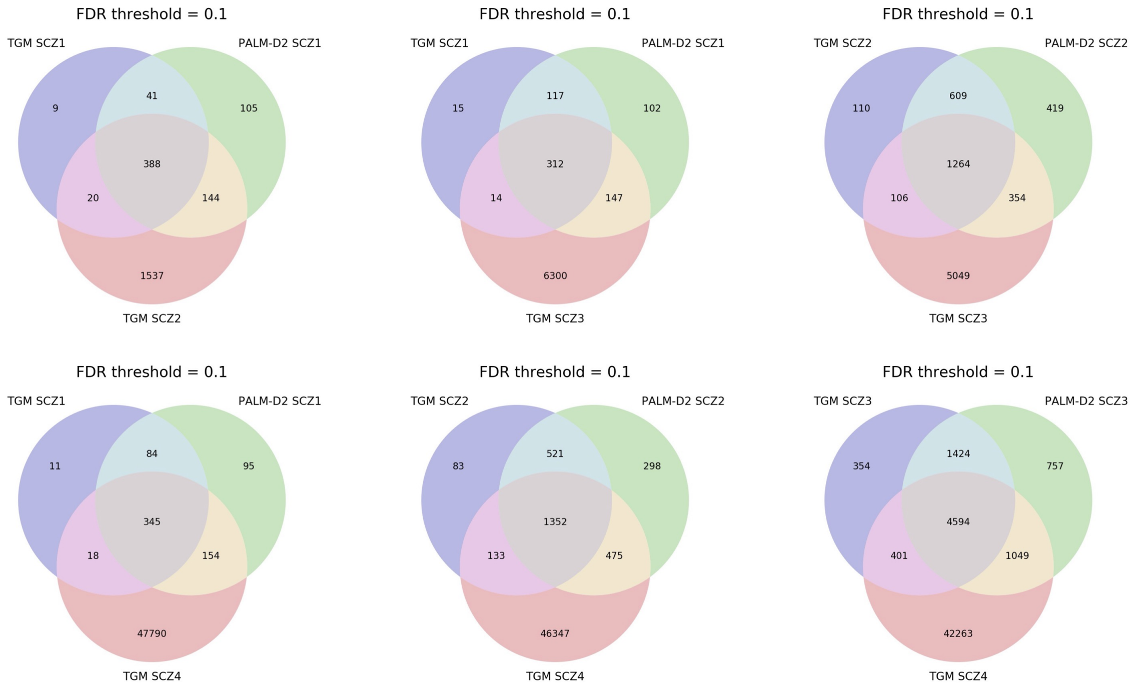

Figure S26: Venn diagrams comparing SNPs prioritized by TGM, PALM-D2 of SCZ GWAS with a smaller sample size and TGM of SCZ GWAS with a larger sample size under the FDR control threshold $\tau = 0.1$.

2.6 Analysis on two Years of Education GWASs with different sample sizes

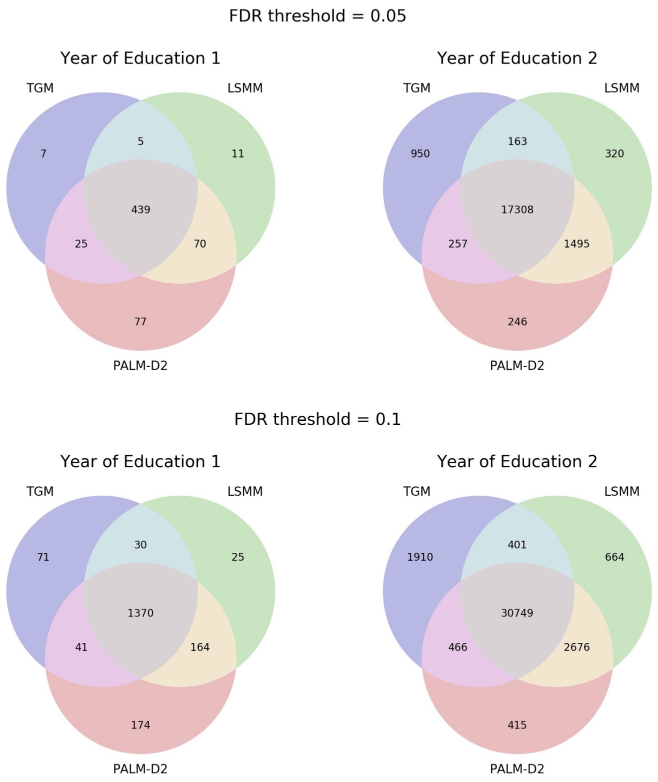

Figure S27: Venn diagrams comparing SNPs prioritized by TGM, LSMM and PALM-D2 of two Years of Education GWASs under the FDR control threshold $\tau = 0.05$ and $\tau = 0.1$.

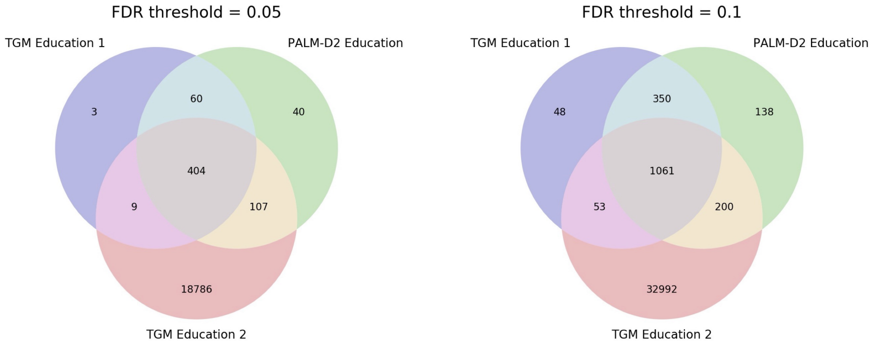

Figure S28: Venn diagrams comparing SNPs prioritized by TGM, PALM-D2 of Years of Education GWAS with a smaller sample size and TGM of Years of Education GWAS with a larger sample size under the FDR control threshold $\tau = 0.05$ and $\tau = 0.1$.

2.7 Sources of 30 GWASs

GWAS/Trait	Sample size	Paper/Data link
Alzheimer	74,046	[13] (https://alkesgroup.broadinstitute.org/sumstats_formatted/)
BMI	249,796	[25] https://alkesgroup.broadinstitute.org/sumstats_formatted/
Bipolar Disorder	16,731	[2] (https://alkesgroup.broadinstitute.org/sumstats_formatted/)
CAD (Coronary Artery Disease)	86,995	[24] (http://www.cardiogramplusc4d.org/data-downloads/)
Crohns Disease	75,000	[11] (https://alkesgroup.broadinstitute.org/sumstats_formatted/)
Height	253,288	[27] (https://portals.broadinstitute.org/collaboration/giant/index.php/GIANT_consortium_data_files)
HDL (High-density Lipoprotein)	188,577	[4] (http://csg.sph.umich.edu//abecasis/public/lipids2013/)
HIV	13,500	[14] (https://journals.plos.org/plospathogens/article?id=10.1371/journal.ppat.1003515)
IBD (Inflammatory Bowel Disease)	75,000	[11] (https://alkesgroup.broadinstitute.org/sumstats_formatted/)
LDL (Low-density Lipoprotein)	188,577	[4] (http://www.sph.umich.edu/csg/abecasis/public/lipids2013/)
Lupus	23,210	[5] (https://www.immunobase.org/downloads/protected_data/GWAS_Data/)
MCH (Mean Cell Haemoglobin)	135,367	[21] (https://ega-archive.org/studies/EGAS00000000132)
MCV (Mean Cell Volume)	135,367	[21] (https://ega-archive.org/studies/EGAS00000000132)
Menopause	69,360	[8] (http://www.reprogen.org/data_download.html)
Multiple Sclerosis	15,474	[1] (https://www.immunobase.org/downloads/protected_data/GWAS_Data/)
Neuroticism	170,911	[18] (http://ssgac.org/documents/Neuroticism_Full.txt.gz)
Primary Biliary Cirrhosis	13,239	[7] (https://www.immunobase.org/downloads/protected_data/GWAS_Data/)
Red Cell Count	135,367	[21] (https://ega-archive.org/studies/EGAS00000000132)
Rheumatoid Arthritis	103,638	[17] (https://alkesgroup.broadinstitute.org/sumstats_formatted/)
SCZ1 (Schizophrenia)	17,115	[16] (https://www.med.unc.edu/pgc/results-and-downloads)
SCZ2 (Schizophrenia)	21,856	[3] (https://www.med.unc.edu/pgc/results-and-downloads)
SCZ3 (Schizophrenia)	32,143	[23] (https://www.med.unc.edu/pgc/results-and-downloads)
SCZ4 (Schizophrenia)	150,064	[20] (https://www.med.unc.edu/pgc/results-and-downloads)
TC (Total Cholesterol)	188,577	[4] (http://csg.sph.umich.edu//abecasis/public/lipids2013/)
TG (Triglycerides)	188,577	[4] (http://csg.sph.umich.edu//abecasis/public/lipids2013/)
Type 1 Diabetes	26,890	[6] (https://www.immunobase.org/downloads/protected_data/GWAS_Data/)
Type 2 Diabetes	149,821	[15] (http://diagram-consortium.org/downloads.html)
Ulcerative Colitis	75,000	[11] (https://alkesgroup.broadinstitute.org/sumstats_formatted/)
Years of Education1	126,559	[22] (https://alkesgroup.broadinstitute.org/sumstats_formatted/)
Years of Education2	293,723	[19] (http://ssgac.org/documents/EduYears_Main.txt.gz)

Table S4: Sources of 30 GWASs in the real data analysis.

References

[1] Genetic risk and a primary role for cell-mediated immune mechanisms in multiple sclerosis. *Nature*, 476(7359):214–219, 2011.

[2] Large-scale genome-wide association analysis of bipolar disorder identifies a new susceptibility locus near ODZ4. *Nature genetics*, 43(10):977–983, 2011.

[3] Genome-wide association study identifies five new schizophrenia loci. *Nature genetics*, 43 (10):969–976, 2011.

[4] Discovery and refinement of loci associated with lipid levels. *Nature genetics*, 45(11): 1274–1283, 2013.

[5] J. Bentham, D. L. Morris, D. S. Cunninghame Graham, C. L. Pinder, P. Tombleson, T. W. Behrens, J. Martín, B. P. Fairfax, J. C. Knight, L. Chen, et al. Genetic association analyses implicate aberrant regulation of innate and adaptive immunity genes in the pathogenesis of systemic lupus erythematosus. *Nature genetics*, 47(12):1457–1464, 2015.

[6] J. P. Bradfield, H.-Q. Qu, K. Wang, H. Zhang, P. M. Sleiman, C. E. Kim, F. D. Mentch, H. Qiu, J. T. Glessner, K. A. Thomas, et al. A genome-wide meta-analysis of six type 1 diabetes cohorts identifies multiple associated loci. *PLoS genetics*, 7(9):e1002293, 2011.

[7] H. J. Cordell, Y. Han, G. F. Mells, Y. Li, G. M. Hirschfield, C. S. Greene, G. Xie, B. D. Juran, D. Zhu, D. C. Qian, et al. International genome-wide meta-analysis identifies new primary biliary cirrhosis risk loci and targetable pathogenic pathways. *Nature communications*, 6(1):1–11, 2015.

[8] F. R. Day, K. S. Ruth, D. J. Thompson, K. L. Lunetta, N. Pervjakova, D. I. Chasman, L. Stolk, H. K. Finucane, P. Sulem, B. Bulik-Sullivan, et al. Large-scale genomic analyses link reproductive aging to hypothalamic signaling, breast cancer susceptibility and brca1-mediated dna repair. *Nature genetics*, 47(11):1294–1303, 2015.

[9] J. H. Friedman. Greedy function approximation: a gradient boosting machine. *Annals of statistics*, pages 1189–1232, 2001.

[10] J. H. Friedman and B. E. Popescu. Predictive learning via rule ensembles. *The annals of applied statistics*, pages 916–954, 2008.

[11] L. Jostins, S. Ripke, R. K. Weersma, R. H. Duerr, D. P. McGovern, K. Y. Hui, J. C. Lee, L. Philip Schumm, Y. Sharma, C. A. Anderson, et al. Host–microbe interactions have shaped the genetic architecture of inflammatory bowel disease. *Nature*, 491(7422):119–124, 2012.

[12] A. Khatiwada, B. J. Wolf, A. S. Yilmaz, P. S. Ramos, M. Pietrzak, A. Lawson, K. J. Hunt, H. J. Kim, and D. Chung. GPA-tree: statistical approach for functional-annotation-tree-guided prioritization of GWAS results. *Bioinformatics*, 38(4):1067–1074, 2022.

[13] J.-C. Lambert, C. A. Ibrahim-Verbaas, D. Harold, A. C. Naj, R. Sims, C. Bellenguez, G. Jun, A. L. DeStefano, J. C. Bis, G. W. Beecham, et al. Meta-analysis of 74,046 individuals identifies 11 new susceptibility loci for alzheimer's disease. *Nature genetics*, 45 (12):1452–1458, 2013.

[14] P. J. McLaren, C. Coulonges, S. Ripke, L. Van Den Berg, S. Buchbinder, M. Carrington, A. Cossarizza, J. Dalmau, S. G. Deeks, O. Delaneau, et al. Association study of common genetic variants and hiv-1 acquisition in 6,300 infected cases and 7,200 controls. *PLoS pathogens*, 9(7):e1003515, 2013.

[15] M.-A. of Glucose, I. related traits Consortium (MAGIC) Investigators, G. I. of ANthropometric Traits (GIANT) Consortium, A. G. E. N.-T. . D. A.-T. Consortium, S. A. T. . D. S. Consortium, A. R. Shuldiner, M. Roden, I. Barroso, T. Wilsgaard, J. Beilby, et al. Large-scale association analysis provides insights into the genetic architecture and pathophysiology of type 2 diabetes. *Nature genetics*, 44(9):981–990, 2012.

[16] C.-D. G. of the Psychiatric Genomics Consortium et al. Identification of risk loci with shared effects on five major psychiatric disorders: a genome-wide analysis. *The Lancet*, 381(9875):1371–1379, 2013.

[17] Y. Okada, D. Wu, G. Trynka, T. Raj, C. Terao, K. Ikari, Y. Kochi, K. Ohmura, A. Suzuki, S. Yoshida, et al. Genetics of rheumatoid arthritis contributes to biology and drug discovery. *Nature*, 506(7488):376–381, 2014.

[18] A. Okbay, B. M. Baselmans, J.-E. De Neve, P. Turley, M. G. Nivard, M. A. Fontana, S. F. W. Meddens, R. K. Linnér, C. A. Rietveld, J. Derringer, et al. Genetic variants associated with subjective well-being, depressive symptoms, and neuroticism identified through genome-wide analyses. *Nature genetics*, 48(6):624–633, 2016a.

[19] A. Okbay, J. P. Beauchamp, M. A. Fontana, J. J. Lee, T. H. Pers, C. A. Rietveld, P. Turley, G.-B. Chen, V. Emilsson, S. F. W. Meddens, et al. Genome-wide association study identifies 74 loci associated with educational attainment. *Nature*, 533:539–542, 2016b.

[20] C. Pantelis, G. N. Papadimitriou, S. Papiol, E. Parkhomenko, M. T. Pato, T. Paunio, M. Pejovic-Milovancevic, D. O. Perkins, O. Pietiläinen, et al. Biological insights from 108 schizophrenia-associated genetic loci. *Nature*, 511(7510):421–427, 2014.

[21] J. K. Pickrell. Joint analysis of functional genomic data and genome-wide association studies of 18 human traits. *The American Journal of Human Genetics*, 94(4):559–573, 2014.

[22] C. A. Rietveld, S. E. Medland, J. Derringer, J. Yang, T. Esko, N. W. Martin, H.-J. Westra, K. Shakhbazov, A. Abdellaoui, A. Agrawal, et al. Gwas of 126,559 individuals identifies genetic variants associated with educational attainment. *science*, 340(6139):1467–1471, 2013.

[23] S. Ripke, C. O'dushlaine, K. Chambert, J. L. Moran, A. K. Kähler, S. Akterin, S. E. Bergen, A. L. Collins, J. J. Crowley, M. Fromer, et al. Genome-wide association analysis identifies 13 new risk loci for schizophrenia. *Nature genetics*, 45(10):1150–1159, 2013.

[24] H. Schunkert, I. R. König, S. Kathiresan, M. P. Reilly, T. L. Assimes, H. Holm, M. Preuss, A. F. Stewart, M. Barbalic, C. Gieger, et al. Large-scale association analysis identifies 13 new susceptibility loci for coronary artery disease. *Nature genetics*, 43(4):333–338, 2011.

[25] E. K. Speliotes, C. J. Willer, S. I. Berndt, K. L. Monda, G. Thorleifsson, A. U. Jackson, H. L. Allen, C. M. Lindgren, J. Luan, R. Mägi, et al. Association analyses of 249,796 individuals reveal 18 new loci associated with body mass index. *Nature genetics*, 42(11): 937–948, 2010.

[26] K. Wang, M. Li, and H. Hakonarson. Annovar: functional annotation of genetic variants from high-throughput sequencing data. *Nucleic acids research*, 38(16):e164–e164, 2010.

[27] A. R. Wood, T. Esko, J. Yang, S. Vedantam, T. H. Pers, S. Gustafsson, A. Y. Chu, K. Estrada, J. Luan, Z. Kutalik, et al. Defining the role of common variation in the genomic and biological architecture of adult human height. *Nature genetics*, 46(11): 1173–1186, 2014.
